# Kinetics of the B- and T-Cell Immune Responses After 6 Months From SARS-CoV-2 mRNA Vaccination in Patients With Rheumatoid Arthritis

**DOI:** 10.3389/fimmu.2022.846753

**Published:** 2022-02-28

**Authors:** Chiara Farroni, Andrea Picchianti-Diamanti, Alessandra Aiello, Emanuele Nicastri, Bruno Laganà, Chiara Agrati, Concetta Castilletti, Silvia Meschi, Francesca Colavita, Gilda Cuzzi, Rita Casetti, Germana Grassi, Linda Petrone, Valentina Vanini, Andrea Salmi, Federica Repele, Anna Maria Gerarda Altera, Gaetano Maffongelli, Angela Corpolongo, Simonetta Salemi, Roberta Di Rosa, Gabriele Nalli, Giorgio Sesti, Francesco Vaia, Vincenzo Puro, Delia Goletti

**Affiliations:** ^1^Translational Research Unit, National Institute for Infectious Diseases Lazzaro Spallanzani-Istituto di Ricovero e Cura a Carattere Scientifico (IRCCS), Rome, Italy; ^2^Department of Clinical and Molecular Medicine, “Sapienza” University, S. Andrea University Hospital, Rome, Italy; ^3^Clinical Division of Infectious Diseases, National Institute for Infectious Diseases Lazzaro Spallanzani-Istituto di Ricovero e Cura a Carattere Scientifico (IRCCS), Rome, Italy; ^4^Laboratory of Cellular Immunology, National Institute for Infectious Diseases Lazzaro Spallanzani-Istituto di Ricovero e Cura a Carattere Scientifico (IRCCS), Rome, Italy; ^5^Laboratory of Virology, National Institute for Infectious Diseases Lazzaro Spallanzani-Istituto di Ricovero e Cura a Carattere Scientifico (IRCCS), Rome, Italy; ^6^Unità Operativa Semplice (UOS) Professioni Sanitarie Tecniche, National Institute for Infectious Diseases Lazzaro Spallanzani-Istituto di Ricovero e Cura a Carattere Scientifico (IRCCS), Rome, Italy; ^7^Unità Operativa Complessa (UOC) Direzione Sanitaria, National Institute for Infectious Diseases Lazzaro Spallanzani-Istituto di Ricovero e Cura a Carattere Scientifico (IRCCS), Rome, Italy; ^8^Unità Operativa Complessa (UOC) Emerging Infections and Centro di Riferimento AIDS (CRAIDS), National Institute for Infectious Diseases Lazzaro Spallanzani-Istituto di Ricovero e Cura a Carattere Scientifico (IRCCS), Rome, Italy

**Keywords:** COVID-19, mRNA vaccine, rheumatoid arthritis, whole blood, T-cell response, antibody response, DMARD (disease-modifying antirheumatic drug), biological therapy

## Abstract

**Objective:**

To assess the kinetics of the humoral and cell-mediated responses after severe acute respiratory syndrome coronavirus 2 (SARS-CoV-2) vaccination in rheumatoid arthritis (RA) patients treated with different immunosuppressive therapies.

**Methods:**

Following vaccine completed schedule, health care workers (HCWs, n = 49) and RA patients (n = 35) were enrolled at 5 weeks (T1) and 6 months (T6) after the first dose of BNT162b2-mRNA vaccination. Serological response was assessed by quantifying anti-receptor-binding domain (RBD)-specific immunoglobulin G (IgG) and SARS-CoV-2 neutralizing antibodies, while cell-mediated response was assessed by a whole-blood test quantifying the interferon (IFN)-γ response to spike peptides. B-cell phenotype and IFN-γ-specific T-cell responses were evaluated by flow cytometry.

**Results:**

After 6 months, anti-RBD antibodies were still detectable in 91.4% of RA patients, although we observed a significant reduction of the titer in patients under Cytotoxic T-Lymphocyte Antigen 4 (CTLA-4)-Ig [median: 16.4 binding antibody units (BAU)/ml, interquartile range (IQR): 11.3–44.3, p < 0.0001] or tumor necrosis factor (TNF)-α inhibitors (median: 26.5 BAU/ml, IQR: 14.9–108.8, p = 0.0034) compared to controls (median: 152.7 BAU/ml, IQR: 89.3–260.3). All peripheral memory B-cell (MBC) subpopulations, in particular, the switched IgG^+^ MBCs (CD19^+^CD27^+^IgD^-^IgM^-^IgG^+^), were significantly reduced in RA subjects under CTLA-4-Ig compared to those in HCWs (p = 0.0012). In RA patients, a significantly reduced anti-RBD IgG titer was observed at T6 vs. T1, mainly in those treated with CTLA-4-Ig (p = 0.002), interleukin (IL)-6 inhibitors (p = 0.015), and disease-modifying antirheumatic drugs (DMARDs) ± corticosteroids (CCSs) (p = 0.015). In contrast, a weak nonsignificant reduction of the T-cell response was reported at T6 vs. T1. T-cell response was found in 65.7% of the RA patients at T6, with lower significant magnitude in patients under CTLA-4-Ig compared to HCWs (p < 0.0001). The SARS-CoV-2 IFN-γ-S-specific T-cell response was mainly detected in the CD4^+^ T-cell compartment.

**Conclusions:**

In this study, in RA patients after 6 months from COVID-19 vaccination, we show the kinetics, waning, and impairment of the humoral and, to a less extent, of the T-cell response. Similarly, a reduction of the specific response was also observed in the controls. Therefore, based on these results, a booster dose of the vaccine is crucial to increase the specific immune response regardless of the immunosuppressive therapy.

## Introduction

Severe acute respiratory syndrome coronavirus 2 (SARS-CoV-2) emerges in 2019, causing the actual COronaVIrus Disease 2019 (COVID-19) pandemic. The clinical manifestations are wide from asymptomatic to mild infection of the upper respiratory tract until the severe respiratory failure that requires intensive care unit hospitalization (1–6).

A program of global vaccination is the most successful strategy to control the COVID-19 pandemic. Evidence is currently available about the efficacy of mRNA vaccines, such as BNT162b2 and mRNA-1273 vaccines, in eliciting strong antibody and cell-mediated immune responses in healthy subjects (7–9).

Recently, promising data, mainly at 1 month from the completion of the vaccine schedule, have demonstrated the immunogenicity and safety of BNT162b2 mRNA vaccine in rheumatoid arthritis (RA) patients (10–12).

Altogether, these studies show that the humoral response to BNT162b2 vaccine is immunogenic in the majority of RA patients, although the response is delayed and decreased compared to controls (10). In particular, we showed that RA patients have a lower quantitative immune response (both antibody- and T cell-specific responses) to BNT162b2 vaccine compared to a control group of health care workers (HCWs), even if the vaccine is qualitatively immunogenic for most RA patients (13).

Recently, it has been shown that the COVID-19 vaccine-induced immunity decreases over time (14–17), and these results promoted a booster vaccination to the population to restore the vaccine efficiency (14, 15). It is unclear if these results are confirmed in RA patients and whether the immune-modulating treatments are associated with an impairment of the B- or T-cell response to vaccine evaluated over time.

Therefore, the aim of the present prospective study was to evaluate the kinetics of both humoral and cell-mediated responses after 5 weeks (T1) and 6 months (T6) from the first dose of SARS-CoV-2 vaccination in RA patients treated with different immunosuppressive therapies who completed the vaccine schedule.

## Methods

### Study Population

**Figure 1:**
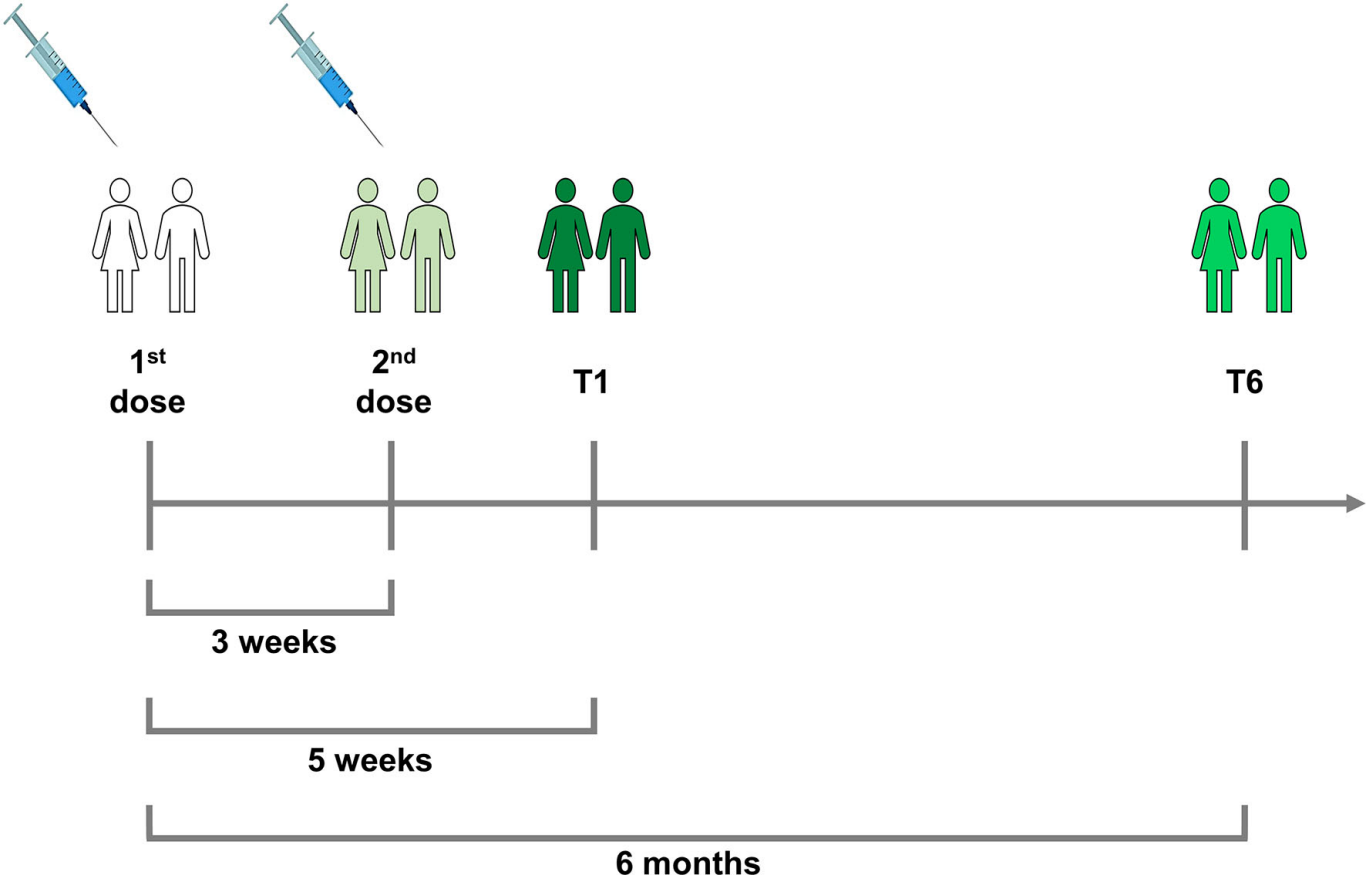
Timeline of the two-dose schedule of BNT162b2 mRNA vaccine and of the enrollment of rheumatoid arthritis (RA) and health care worker (HCW) individuals. All subjects received the second vaccine dose after 3 weeks from the first one. They were enrolled after 5 weeks (T1) and after 6 months from the first dose of vaccine (T6). At T1 and T6, blood samples were collected from all the participants.

HCWs were prospectively enrolled at the National Institute for Infectious Diseases (INMI) Lazzaro Spallanzani-IRCCS (Approval number 297/2021). Patients with a diagnosis of RA according to the 2010 criteria of the European League Against Rheumatism/American College of Rheumatology (EULAR/ACR) (18) were enrolled at Sant’Andrea University Hospital in Rome (Approval number 318/2021). Participants were recruited according to the following criteria: having received two doses of BNT162b2 mRNA vaccine and be longitudinally sampled after 5 weeks (T1) and 6 months (T6) from the first dose (**Figure 1**). The enrolled RA patients were under treatment with a biological drug with or without methotrexate (MTX) or other disease-modifying antirheumatic drugs (DMARDs; i.e., sulfasalazine and leflunomide), with only DMARDs, or low dosage of corticosteroids (CCSs) (prednisone <7.5 mg/day or equivalent). RA subjects treated with anti-CD20 or anti-Janus kinase (JAK) were excluded due to the small number of recruited individuals. Additional exclusion criterion was recent or remote SARS-CoV-2 infection. For the enrollment, both HCWs and RA patients signed a written informed consent.

### Study Design

Demographic and clinical data were collected. We assessed the RA disease activity through the Disease Activity Score based on C-reactive protein (DAS28crp). Blood was collected in heparin tubes; the presence of any clinical adverse events was registered. RA patients were divided into four groups according to drug treatment: TNF-α inhibitors with or without DMARDs (hereafter referred to as TNF-α inhibitors), interleukin (IL)-6 inhibitors with or without DMARDs/CCSs (hereafter referred to as IL-6 inhibitors), CTLA-4-Ig with or without DMARDs/CCSs (hereafter referred to as CTLA-4-Ig), and DMARDs with or without CCSs (hereafter referred to as DMARDs). Moreover, within a week from blood collection, the lymphocyte count of the RA patients was performed. As previously described (19), the ongoing therapeutic regimen was modified during the vaccination period according to the ACR indications (18). In particular, MTX was interrupted for 1 week after the first and second doses, whereas abatacept (CTLA-4-Ig) was stopped 1 week before and after the first dose only.

### Anti-SARS-CoV-2-Specific IgG Evaluation

The humoral response to vaccination was measured as previously reported (20). The assays detect both the anti-nucleoprotein-immunoglobulin G (Anti-N-IgG) and the anti-RBD-IgG (Architect^®^ i2000sr Abbott Diagnostics, Chicago, IL, USA). Anti-N-IgG is indicated as index value (S/CO) that was considered positive if ≥1.4, while anti-RBD-IgG was expressed as binding antibody units (BAU)/ml and indicated as positive when ≥7.1.

### Micro-Neutralization Assay

To evaluate the levels of neutralizing antibodies to SARS-CoV-2, a micro-neutralization assay (MNA) was performed as described (21) using the live SARS-CoV-2 virus (strain 2019-nCoV/Italy-INMI1; GISAID accession ID: EPI_ISL_412974). The assay evaluates the cytopathic effect (CPE) at 48 h post infection through the inhibition of Vero E6 cell infection by serum dilution curves. Microplates were observed by light microscope for the presence of CPE and, then stained with crystal violet solution containing 2% formaldehyde. Cell viability was measured by photometer at 595 nm (Synergy™ HTX Multi-Mode Microplate Reader, Agilent Biotek, Milan, Italy). Neutralization titer was expressed as the reciprocal of serum dilution (MNA_90_) and corresponds to the highest serum dilution that inhibited at least 90% of the CPE.

### B-Cell Phenotype

B-cell evaluation was performed using the B-cell Tubes (22) according to the manufacturer’s procedure (BD Biosciences, San Jose, California (CA), USA) (see **Supplementary Table S1** for antibodies and reagents). Briefly, freshly collected whole blood (300 µl) (HCWs, n = 7; RA, n = 13) was washed three times in a 15-ml tube with 10 ml of FACS Buffer (BD Biosciences, San Jose, USA) to eliminate the IgG present in the serum. Supernatant was removed carefully, and 200 µl of blood was transferred in the B-cell Tube, where lyophilized antibodies were present (CD45, CD19, CD24, CD27, CD38, IgD, IgM, IgG). Each tube was mixed gently for 3–5 s and then incubated for 20 min at room temperature (RT) in the dark. Afterward, 4 ml of BD Lysing solution (BD Biosciences, San Jose, USA) were added to lyse red blood cells for 15 min at RT. Finally, cells were spinned down for 5 min at 200 × g and resuspended in 300 µl of FACS Buffer. Samples were acquired on a BD Lyric cytometer (BD Biosciences, San Jose, USA), and FlowJo software (version 10.8.1, Tree Star) was used to analyze the data (see **Supplementary Figure S1** for the gating strategy).

### IFN-γ Whole-Blood Assay

To evaluate the specific IFN-γ-Spike (S)-specific T-cell response, a whole-blood platform was used (20, 23–29). We stimulated the whole blood with a pool of peptides that covered the SARS-CoV-2-S protein sequence (SARS-CoV-2 PepTivator^®^ Prot_S1, Prot_S, and Prot_S+, cat. 130-127-048, cat. 130-126-701, and cat. 130-127-312, respectively, Miltenyi Biotec, Germany), consisting of 15-amino acid length with an 11-amino acid overlap.

Briefly, 600 µl of whole blood were *in vitro* stimulated for 24 h at 37°C (5% CO_2_) with SARS-CoV-2 PepTivator^®^ Peptide Pools in a 48-well flat-bottom plate, as described (23). Whole blood was also stimulated with staphylococcal enterotoxin B (SEB) (Merck Life Science Cat. S4881) at 200 ng/ml as positive control. After 20–24 h, plasma was harvested and stored at -80°C until further use. The IFN-γ levels were quantified by an automatic ELISA (ELLA, protein simple, R&D Systems, Minneapolis, MN, USA, cat. SPCKB-PS-002574), and the values obtained were subtracted from the unstimulated control. The detection limit of the assay was 0.17 pg/ml. IFN-γ values ≥16 pg/ml were considered positive based on a cutoff found by Receiver Operating Characteristic (ROC) analysis comparing the response in COVID-19 patients vs. no-COVID individuals (article in preparation).

Samples used for the intracellular evaluation of the IFN-γ production were also costimulated with α-CD28 and α-CD49d monoclonal antibodies (BD Biosciences, San Jose, USA), as described below.

### Functional Analysis by Intracellular Staining and Flow Cytometry

To assess if the T-cell subpopulations CD4 and CD8 mount a SARS-CoV-2-S-specific response, we analyzed the IFN-γ-S-specific T-cell frequency by flow cytometry. To this aim, cells from whole blood were stimulated with the spike peptide pool and then fixed before the flow cytometry analysis. Briefly, spike peptide pool (0.1 μg/ml) was used to stimulate whole blood from RA and HCWs for 24 h with α-CD28 together with α-CD49 (1 μg/ml each). After collecting plasma, whole blood was cultured for another 5 h in the presence of brefeldin A (1 μg/ml) (cat. B7450, Life Technologies, Monza, Italy) to inhibit cytokine secretion. Then, blood was harvested and stained with Fixable Viability stain 700 (BD Biosciences, San Jose, USA) for 10 min at RT protected from light. Red blood cells were lysed with BD Lysing Solution (BD Biosciences, San Jose, USA) 1X+ 4% of formaldehyde for 10 min at RT, then cells were washed with 1 ml of Phosphate Buffered Saline (PBS) and centrifuged at 600 × g for 5 min. Cells were fixed with 4% formaldehyde for 5 min, washed again with 1 ml of PBS, and centrifuged at 600 × g for 5 min. At the end of the procedure, cells were frozen in Fetal Calf Serum (FCS) + 10% Dimethyl Sulfoxide (DMSO) until further analysis (see **Supplementary Table S1** for the complete list of reagents and stimuli used for flow cytometry analysis) (30, 31). Stimulated and fixed cells were thawed at 37°C, washed twice with PBS 1X at 600 × g for 5 min and transferred to a 96-well round plate (COSTAR, Sigma Aldrich, Milan, Italy) to proceed with the staining procedures. Here, 100 μl/sample of Perm/Wash 1X (BD Biosciences, San Jose, USA) were added for 10 min at RT to permeabilize cells. Subsequently, cells were washed in PBS 1X for 5 min at 600 × g. Then, cells were stained for the surface and intracellular markers, prepared in Brilliant Stain Buffer (BD Biosciences, San Jose, USA) (see **Supplementary Table S1** for the complete list of antibodies and reagents). After 1 h at 4°C, samples were washed twice in Perm/Wash 1X (30, 31). Samples were acquired on a BD Lyric cytometer (BD Biosciences, San Jose, USA), and the analyses were performed with FlowJo software (version 10.8.1, Tree Star) and stratified according to the drug treatment (see **Supplementary Figures S2A, B** for the gating strategy). We considered as positive an IFN-γ-S-specific T-cell response if the percentage of the SARS-CoV-2 peptide-stimulated cells was at least 2-fold higher compared to that of the unstimulated control and if a minimum of 10 events were present within the cytokine gate (32).

### Statistical Analysis

GraphPad software (GraphPad Prism 8 XML ProjecT) was used to analyze the results. The continuous variables IFN-γ levels and anti-RBD and MNA_90_ titers were reported as median and interquartile range (IQR), while categorical variables were stated as count and proportion. The following non-parametric statistical inference tests were used: the Kruskal–Wallis test for comparisons among groups; the Mann–Whitney U-test and Wilcoxon test for pairwise comparisons (for unpaired and paired data, respectively). Bonferroni correction was applied when appropriate. Categorical variables were analyzed by the chi-square test. Correlations between assays were evaluated by non-parametric Spearman’s rank test. A Spearman’s rho >0.7 indicated a high correlation, 0.7 > rho > 0.5 indicated a moderate one, and rho <0.5 indicated a low correlation. Two-tailed p-values were considered significant if <0.05, except for subgroup analyses of RA patients, where a Bonferroni correction was applied with a significant two-tailed p-value threshold of 0.0125 (α/4) or 0.025 for the B-cell analysis (α/2).

## Results

### Demographic and Clinical Characteristics of the Enrolled Subjects

We prospectively enrolled 84 vaccinated individuals: 35 RA patients and 49 HCWs. Significant differences were found for age (p < 0.0001) but not for sex or origin between the two groups (**Table 1**).

**Table 1:** Demographical and clinical characteristics of the 84 enrolled subjects at T6.

Characteristics		RA patients	Health care workers	p-value
N (%)		35 (41.7)	49 (58.3)	
Age median (IQR)		59 (55–66)	51 (45–56)	<0.0001*
Female N (%)		28 (80)	39 (79.6)	0.963^§^
Origin N (%)	West Europe	31 (88.6)	48 (98)	0.288^§^
	East Europe	2 (5.8)	1 (2)	
	Africa	1 (2.8)	0 (0)	
	Sud America	1 (2.8)	0 (0)	
Rheumatologic Treatment N (%)	TNF-α inhibitors ± DMARD	5 (14.3)	–	
	IL-6 inhibitors ± DMARD/CCS	8 (22.9)	–	
	CTLA-4-Ig ± DMARD/CCS	11 (31.4)	–	
	DMARD ± CCS	11 (31.4)	–	
Disease activity median (IQR)	DAS28crp T6	3.3 (2.4–4.0)	–	
Therapy	Years	5.4 (2.5–10.3)	–	
Lymphocytes count N (%)		32 (91.4)	0 (0)	
Lymphocytes count N (%) Median ×10^3^/µl (IQR)	TNF-α inhibitors ± DMARD	5 (15.6)	–	0.276^#^
		2.5 (2.2–3.8)		
	IL-6 inhibitors ± DMARD/CCS	8 (25)	–	
		1.9 (1.5–2.5)		
	CTLA-4-Ig ± DMARD/CCS	11 (34.4)	–	
		2.4 (1.8–2.9)		
	DMARD ± CCS	8 (25)	–	
		2.2 (1.6–2.5)		

DMARDs, disease-modifying antirheumatic drugs; CCS, corticosteroid; RA, rheumatoid arthritis; DAS28, Disease Activity Score 28; N, number; IQR, interquartile range.

*Mann–Whitney U-statistic test.

^§^Chi-square test.

^#^Kruskal–Wallis test.

The RA enrolled cohort was stratified according to the drug treatment: 5 patients were under TNF-α inhibitors, 8 were under IL-6 inhibitors, 11 were under CTLA-4-Ig, and 11 were under DMARDs only. At vaccination, the median treatment duration for TNF-α inhibitors was 3.3 years (IQR: 2.0–13.8), for IL-6 inhibitors 6.4 years (IQR: 5.3–9.8), for CTLA-4-Ig 6.3 years (IQR: 2.2–10.3), and for DMARDs 5.3 years (IQR: 2.6–8.3). Non-serious adverse events to vaccine were reported by either RA patients or HCWs, such as pain at the injection site, headache, or mild fever. Moreover, no serious adverse events (i.e., requiring hospitalization, resulting in death, or life threatening) were reported in vaccinated individuals.

### Humoral Response Persists 6 Months After SARS-CoV-2 Vaccination in Health Care Workers and Rheumatoid Arthritis Patients

**Figure 2:**
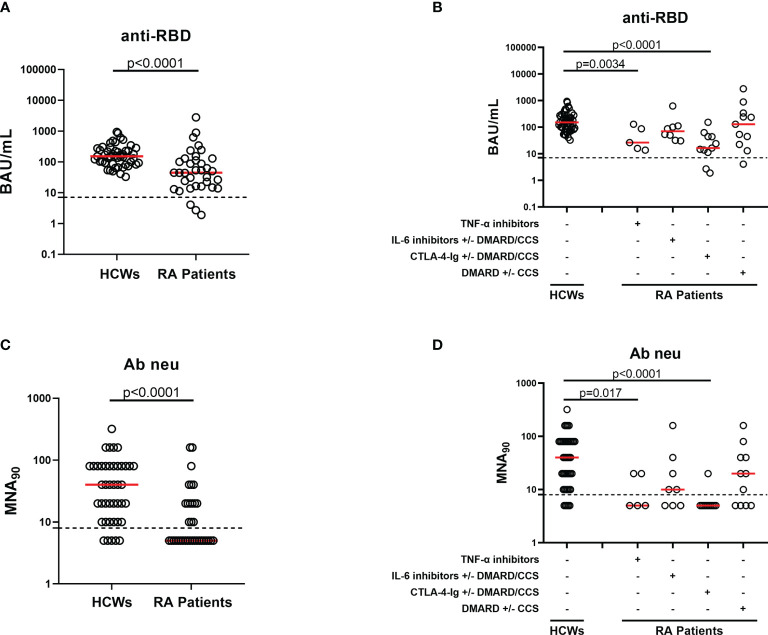
Humoral response elicited by SARS-CoV-2 vaccination after 6 months in HCWs and RA patients. Evaluation of SARS-CoV-2-specific anti-RBD **(A, B)** and neutralizing **(C, D)** antibodies 6 months after vaccination in the total of HCWs (n = 49) and RA patients (n = 35) analyzed. **(B, D)** RA patients were divided according to drug treatment into four groups: TNF-α inhibitors (n = 5), IL-6 inhibitors with or without DMARD/CCS (n = 8), CTLA-4-Ig with or without DMARD/CCS (n = 11), and DMARD with or without CCS (n = 11). Anti-RBD **(A, B)** and neutralizing **(C, D)** antibodies were quantified in serum samples and expressed as binding antibody units (BAU)/ml and reciprocal of dilution (MNA_90_), respectively. Medians were indicated by red horizontal lines, and dashed lines represent the cutoff of each test (anti-RBD: 7.1 BAU/ml and MNA_90_: 8). Mann–Whitney U-test with Bonferroni correction (p ≤ 0.0125) was used for the statistical analysis. SARS-CoV-2, severe acute respiratory syndrome coronavirus 2; CCS, corticosteroid; DMARDs, disease-modifying antirheumatic drugs; RA, rheumatoid arthritis; RBD, receptor-binding domain; HCWs, health care workers.

**Figure 3:**
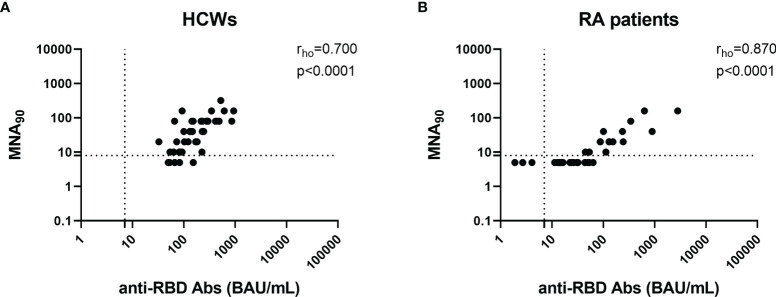
Anti-RBD and neutralizing antibodies correlate with each other in both HCWs and RA patients. Correlation between anti-RBD-IgG titers and neutralizing antibodies in 42 HCWs **(A)** and 35 RA patients **(B)**. Anti-RBD and neutralizing antibodies were quantified in serum samples and expressed as binding antibody units (BAU)/ml and reciprocal of dilution (MNA_90_), respectively. Dashed lines identify the cutoff of each test (anti-RBD: 7.1 BAU/ml and MNA_90_: 8). Statistical analyses were performed using Mann–Whitney U-test with Bonferroni correction (p ≤ 0.0125). Correlation between assays was assessed by non-parametric Spearman’s rank test, and p < 0.05 was considered significant. CCS, corticosteroid; DMARDs, disease-modifying antirheumatic drugs; RA, rheumatoid arthritis; RBD, receptor-binding domain; HCWs, health care workers.

We evaluated the humoral response by assessing the anti-RBD IgG and neutralizing antibodies. All enrolled individuals were negative for anti-N antibodies, confirming that the cohort was naive for SARS-CoV-2 (data not shown). All HCWs (49, 100%) and most RA subjects (32/35, 91.4%) showed a detectable anti-RBD-IgG response after 6 months from vaccination (**Figure 2A** and **Table 2**). However, RA patients under TNF-α inhibitors and CTLA-4-Ig showed a significant lower anti-RBD IgG median titer compared to HCWs (p = 0.0034 and p < 0.0001, respectively) (**Figure 2B**). By contrast, we did not find significant differences for the anti-RBD antibody response in IL-6 inhibitors or DMARD-treated individuals (**Table 2** and **Figure 2B**). We also evaluated the neutralization activity in the sera of all RA patients and in 42/49 HCWs (**Figure 2C**). We found that the majority of HCWs (37/42, 88%), but only 15/35 (42.8%) RA patients, showed detectable neutralizing antibodies. In particular, a neutralizing activity was present in 2/5 (40%) patients treated with TNF-α inhibitors, 5/8 (62.5%) patients under IL-6 inhibitors, 1/11 (9.1%) under CTLA-4-Ig, and 7/11 (63.6%) DMARD-treated patients. Moreover, a significant lower neutralizing titer was observed in patients treated with TNF-α inhibitors or CTLA-4-Ig compared to HCWs (p = 0.017 and p < 0.0001, respectively) (**Figure 2D**). A strong significant correlation was observed between the neutralizing antibody and anti-RBD IgG titers in both HCWs (rho = 0.700, p < 0.0001) and RA patients (rho = 0.870, p < 0.0001) (**Figures 3A, B****)**, while there was no correlation between the number of lymphocytes and the anti-RBD antibody titer (rho = -0.234, p = 0.198) (data not shown).

**Table 2:** Serological and T-cell specific response at T6.

Characteristics				RA patients	Health care workers	p-value	
			**N (%)**	35 (41.7)	49 (58.3)		
**Antibody response**	**Qualitative response**	**Anti-RBD Ab responders**, **N (%)**		32 (91.4)	49 (100)		0.069^§^
		**Anti-RBD Ab responders within the subgroups**,**N (%)**	**TNF-α inhibitors**	5/5 (100)	–	0.641^§^	>0.999^§^
			**IL-6 inhibitors± DMARD/CCS**	8/8 (100)	–		>0.999^§^
			**CTLA-4-Ig± DMARD/CCS**	9/11 (81.8)	–		0.031^§^
			**DMARD±CCS**	10/11 (90.9)	–		0.183^§^
	**Quantitative response**	**Anti-RBD Abs**, **BAU/ml Median (IQR)**		44.9 (16.1–129.3)	152.7 (89.3–260.3)		<0.0001*
			**TNF-α inhibitors**	26.5 (14.9–108.8)	–	0.054^#^	0.0034*
			**IL-6 inhibitors± DMARD/CCS**	70.4 (36.6–109.6)	–		0.023*
			**CTLA-4-Ig± DMARD/CCS**	16.4 (11.3–44.3)	–		<0.0001*
			**DMARD±CCS**	129.3 (22.5–342.3)	–		0.521*
**Spike-specific IFN-γ T-cell response**	**Qualitative response**	**Anti-S responders, N (%)**		23 (65.7)	48 (97.9)		<0.0001^§^
		**Anti-S responders within the subgroups**, **N (%)**	**TNF-α inhibitors± DMARD**	4/5 (80)	–	0.007^§^	0.178^§^
			**IL-6 inhibitors± DMARD/CCS**	8/8 (100)	–		>0.999^§^
			**CTLA-4-Ig± DMARD/CCS**	3/11 (27.3)	–		**<0.0001^§^**
			**DMARD±CCS**	8/11 (72.7)	–		0.017^§^
	**Quantitative response**	**Anti-S IFN-γ**, **pg/ml Median (IQR)**		45.3 (6.3–121.4)	199.5 (81.8–310.8)		<0.0001*
			**TNF-α inhibitors± DMARD**	83.1 (27.7–268.4)	–	0.033^#^	0.172*
			**IL-6 inhibitors± DMARD/CCS**	133.7 (22.6–241.2)	–		0.197*
			**CTLA-4-Ig± DMARD/CCS**	6.3 (1.2–23.6)	–		<0.0001*
			**DMARD±CCS**	87.8 (9.5–121.4)	–		0.022*

DMARDs, disease-modifying antirheumatic drugs; CCS, corticosteroid; RA, rheumatoid arthritis; N, number; IQR, interquartile range; Abs, antibodies; RBD, receptor-binding-domain; S, spike.

^§^Chi-square test.

*Mann–Whitney U-statistic test.

^#^Kruskal–Wallis test.

In bold are only those values that were significant after multiplicity correction by the Bonferroni method (α/4=0.0125).

### Decay of the Antibody Response From T1 to T6 in Health Care Workers and Rheumatoid Arthritis Patients

**Figure 4:**
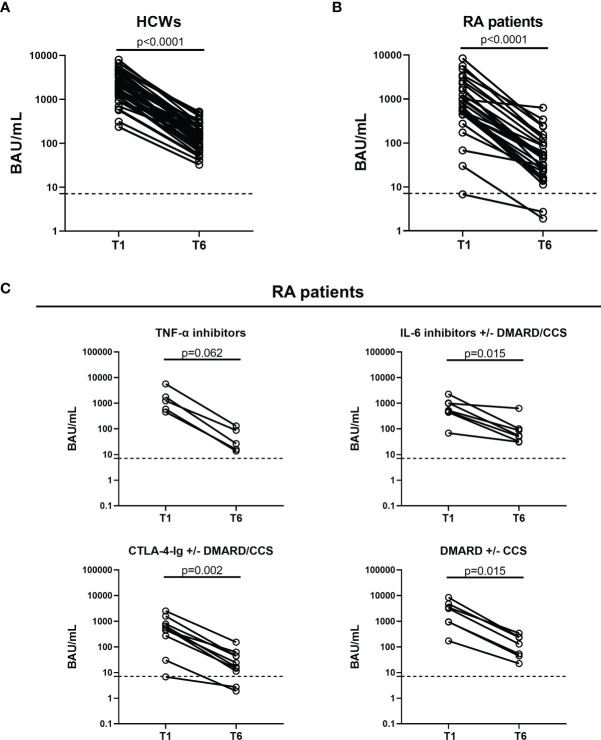
Kinetics of the humoral response induced by SARS-CoV-2 vaccination in HCWs and RA patients. Evaluation of the humoral response in 42 HCWs **(A)** and 29 RA patients **(B)** who were longitudinally sampled after 5 weeks (T1) and 6 months (T6) from the first vaccine dose. **(C)** RA patients were stratified based on the drug treatment: TNF-α inhibitors (n = 5), IL-6 inhibitors with or without DMARD/CCS (n = 7), CTLA-4-Ig with or without DMARD/CCS (n = 10), and DMARD with or without CCS (n = 7). Anti-RBD antibodies were quantified in serum samples and expressed as binding antibody units (BAU)/ml. Dashed lines indicate the cutoff of the test (anti-RBD: 7.1 BAU/ml). Statistical analysis was performed using Wilcoxon test, and p < 0.05 was considered significant. SARS-CoV-2, severe acute respiratory syndrome coronavirus 2; CCS, corticosteroid; DMARDs, disease-modifying antirheumatic drugs; RA, rheumatoid arthritis; RBD, receptor-binding domain; HCWs, health care workers.

To assess the decay of the humoral response to BNT162b2-mRNA vaccine, 42 (85.7%) HCWs and 29 (82.8%) RA patients were longitudinally sampled at T1 and T6. Among the RA patients, 5 subjects under treatment with TNF-α inhibitors, 7 under IL-6 inhibitors, 10 under CTLA-4-Ig, and 7 under DMARDs were followed up. The anti-RBD IgG titer was reduced at T6 compared to T1 in both HCWs (T1 median: 1,891 BAU/ml, IQR: 1,314–3,794 vs. T6 median: 149.4 BAU/ml, IQR: 82.35–226.8, p < 0.0001) (**Figure 4A**) and RA patients (T1 median: 784.7 BAU/ml, IQR: 448.8–2,006 vs. T6 median: 44.9 BAU/ml, IQR: 19.5–115.2, p < 0.0001) (**Figure 4B**). Among RA patients, a significantly reduced titer was observed mainly in those treated with IL-6 inhibitors (T1 median: 518.9 BAU/ml, IQR: 441.4–1,016 vs. T6 median: 53.5 BAU/ml, IQR: 32.1–101, p = 0.015), CTLA-4-Ig (T1 median: 507.3 BAU/ml, IQR: 212.3–986.9 vs. T6 median: 20.3 BAU/ml, IQR: 9.1–49, p = 0.002), and DMARDs ± CCSs (T1 median: 3,170 BAU/ml, IQR: 942.9–4,797 vs. T6 median: 129.3 BAU/ml, IQR: 44.9–244.7, p = 0.015) (**Figure 4C**). An almost significant decrease of the antibody titer was also observed in RA patients treated with TNF-α inhibitors (T1 median: 1,239 BAU/ml, IQR: 520.1–3,706 vs. T6 median: 26.5 BAU/ml, IQR: 14.9–108.8, p = 0.062).

### Memory B Cells Are Reduced in Rheumatoid Arthritis Patients Compared to Health Care Workers at T6

To assess the B-cell compartment of RA patients likely to explain the reduced antibody response at T6, we evaluated the B-cell subpopulation by flow cytometry. We did not observe significant differences between RA patients and HCWs in terms of percentage of total B cells, naïve B cells, transitional B cells, and plasma blasts (**Supplementary Figures S3A–D**).

**Figure 5:**
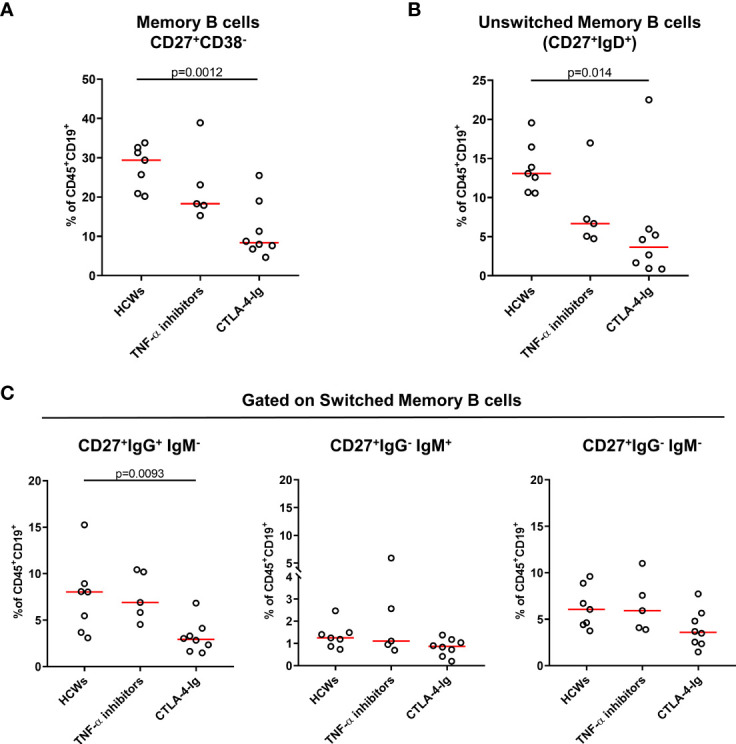
Evaluation of memory B-cell phenotype of RA patients by flow cytometry. Frequency of memory B cells of HCWs (n = 7) and RA patients (n = 13) was evaluated by flow cytometry. Within RA patients, 5 subjects were treated with TNF-α inhibitors and 8 with CTLA-4-Ig. Frequency of **(A)** memory B cells (CD27^+^CD38^-^), subdivided also as **(B)** unswitched memory B cells (CD27^+^IgD^+^) and **(C)** switched memory B cells, is shown. Within the switched memory B cells, CD27^+^IgG^+^IgM^-^, CD27^+^IgG^-^IgM^+^, and CD27^+^IgG^-^IgM^-^ cells were included. Values are reported as percentage of total CD19^+^CD45^+^ B cells. Each dot represents an individual, and the red horizontal line represents medians. Mann–Whitney U-test with Bonferroni correction (p ≤ 0.025) was used for the statistical analysis. RA, rheumatoid arthritis; HCWs, health care workers.

Memory B cells (MBCs) (CD27^+^CD38^-^) were significantly reduced in subjects under CTLA-4-Ig compared to those in HCWs (CTLA-4-Ig median: 8.36%, IQR: 7.0–17.1 vs. HCW median: 29.4%, IQR: 20.9–32.6, p = 0.0012) (**Figure 5A**). In detail, a significant reduction in the unswitched MBCs (CD27^+^IgD^+^) (CTLA-4-Ig median: 3.6%, IQR: 1.1–5.8 vs. HCW median: 13.1%, IQR: 10.7–16.5, p = 0.014) **(****Figure 5B**) and in the switched MBCs (CD27^+^IgD^-^) (CTLA-4-Ig median: 8.0%, IQR: 4.6–9.7 vs. HCW median: 14.7%, IQR: 11.0–19.2, p = 0.0033) was observed (**Supplementary Figure S3E**). Looking at the switched compartment (CD27^+^IgG^+^IgM^-^ or CD27^+^IgG^-^IgM^+^ or CD27^+^IgG^-^IgM^-^), a significant reduction was observed in the IgG^+^ switched MBCs of only CTLA-4-Ig-treated patients compared to those of HCWs (CTLA-4-Ig median: 2.9%, IQR: 1.8–3.9 vs. HCW median: 8.0%, IQR: 3.7–8.9, p = 0.0093) (**Figure 5C**, left panel). We did not find significant differences comparing the results from the patients under TNF-α inhibitor-treated subjects with the results from the controls (**Figures 5A–C** and **Supplementary Figures S3A–D**). Regarding the B-cell subpopulations and memory cell compartment, we did not find significant differences analyzing the results from patients treated with IL-6 inhibitors or DMARDs compared to those from the HCWs (data not shown).

### SARS-CoV-2-Spike-Specific T-Cell Response at T6 in Health Care Workers and Rheumatoid Arthritis

**Figure 6:**
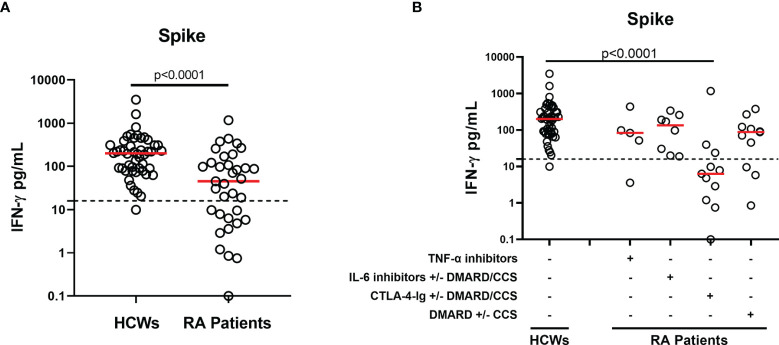
T-cell response after SARS-CoV-2 vaccination in HCWs and RA patients. **(A)** Evaluation of IFN-γ-spike-specific T-cell response 6 months after vaccination in the total of HCWs (n = 49) and RA patients (n = 35). **(B)** RA patients were stratified based on drug treatment in four groups: TNF-α inhibitors (n = 5), IL-6 inhibitors with or without DMARD/CCS (n = 8), CTLA-4-Ig with or without DMARD/CCS (n = 11), and DMARD with or without CCS (n = 11). T-cell response to spike antigen was assessed by measuring IFN-γ levels in plasma harvested from stimulated whole-blood samples. The reported IFN-γ values were subtracted from the background. Dashed lines identify the cutoff of the test (spike: 16 pg/ml). The red horizontal lines indicate the median. Statistical analyses were performed using Mann–Whitney U-test with Bonferroni correction (p ≤ 0.0125). SARS-CoV-2, severe acute respiratory syndrome coronavirus 2; CCS, corticosteroid; DMARDs, disease-modifying antirheumatic drugs; RA, rheumatoid arthritis; HCWs, health care workers.

All HCWs, but one, showed an IFN-γ-S-specific response (48/49, 97.9%), whereas in RA patients a significantly different proportion of responders and a lower quantitative IFN-γ response was observed compared to controls (23/35, 65.7%, p < 0.0001) (**Table 2** and **Figure 6A**). In particular, patients under CTLA-4-Ig showed the lowest number of responders (3/11, 27.3%) (**Table 2**) associated with significantly lower IFN-γ-S-specific levels compared to HCWs (p < 0.0001) (**Figure 6B**). Differently, no significant differences were found between the IFN-γ-S-specific response of RA subjects under TNF-α inhibitors, IL-6 inhibitors, or DMARDs compared to that of HCWs (p = 0.172, p = 0.197, and p = 0.022, respectively). Most RA patients showed a positive response to SEB stimulus, used as positive control. However, the magnitude of the response was significantly lower compared to that of HCWs (RA median: 3,163 pg/ml, IQR: 1,598–6,698 vs. HCW median: 6,172 pg/ml, IQR: 3,422–9,447, p = 0.0027) (**Supplementary Figure S4A**). In detail, patients under TNF-α inhibitors and DMARDs showed the lowest response to SEB stimulus (TNF-α median: 1,170 pg/ml, IQR: 889–4,380, p = 0.011 vs. DMARD median: 2,918 pg/ml, IQR: 1,598–4,301, p = 0.006) **(****Supplementary Figure S4B**).

**Figure 7:**
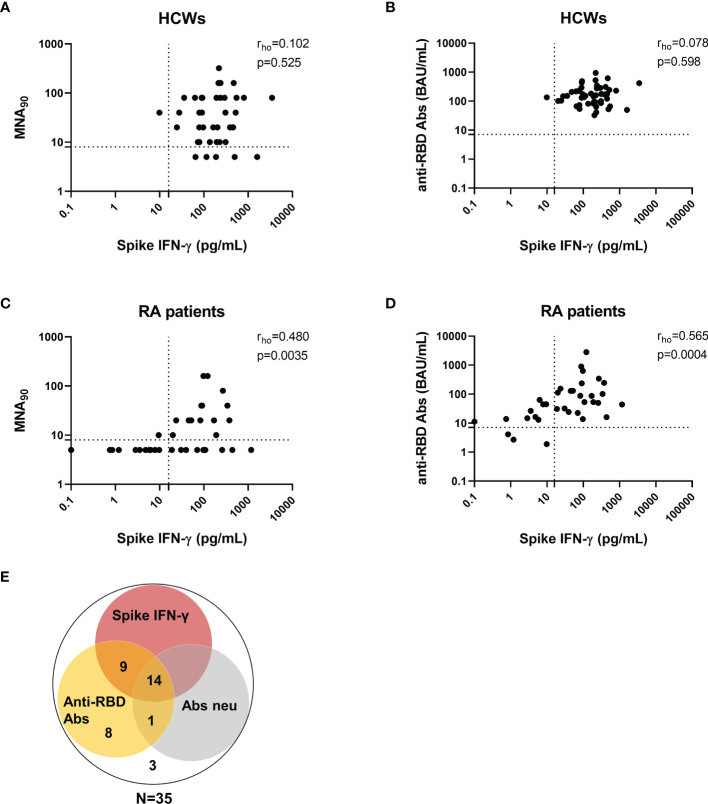
Correlation between T-cell response and anti-RBD or neutralizing antibodies in HCWs and RA patients. Correlation between neutralizing antibodies and the IFN-γ-spike-specific T-cell response in HCWs **(A)** and RA subjects **(C)**. Correlation between anti-RBD antibodies and the IFN-γ-spike-specific T-cell response in HCWs **(B)** and RA subjects **(D)**. Anti-RBD and neutralizing antibodies were quantified in serum samples and expressed as binding antibody units (BAU)/ml and reciprocal of dilution (MNA_90_), respectively. T-cell response to spike antigen was assessed by measuring IFN-γ levels and reported after subtracting the background. Dashed lines identify the cutoff of each test (anti-RBD: 7.1 BAU/ml; MNA_90_: 8 and spike: 16 pg/ml). Correlation between assays was assessed by non-parametric Spearman’s rank test (p < 0.05). **(E)** Venn diagram shows positive results with anti-RBD IgG, neutralizing antibodies and IFN-γ T-cell response. RA, rheumatoid arthritis; RBD, receptor-binding domain; HCWs, health care workers.

We then evaluated the correlation between the B- and T-cell arms of the immune response. No significant correlations between SARS-CoV-2 IFN-γ-S-specific response and neutralizing or anti-RBD antibody titer were observed in HCWs (rho = 0.102, p = 0.525 and rho = 0.078, p = 0.598, respectively) (**Figures 7A, B****)**. By contrast, moderate significant correlations were found in RA patients between the spike IFN-γ response and neutralizing antibodies (rho = 0.480, p = 0.0035) or anti-RBD antibodies (rho = 0.565, p = 0.0004) (**Figures 7C, D****)**. Overall, among RA patients, 14 were full responders (neutralizing activity, anti-RBD antibody and T-cell responses), 3 were not responders, and 18 were partial responders. Among these, 9 scored positive for both spike IFN-γ- and anti-RBD antibody responses, 8 for only anti-RBD antibodies, and 1 subject showed a neutralizing and anti-RBD antibody response (**Figure 7E**). There was no correlation between the number of lymphocytes and the IFN-γ-S-specific response (rho = 0.082, p = 0.654) (data not shown).

**Figure 8:**
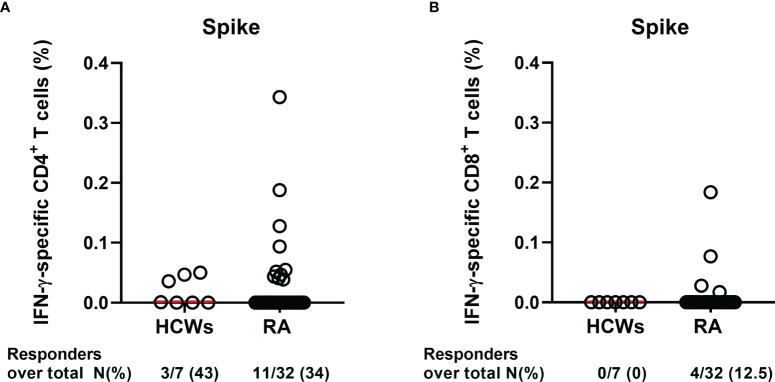
Evaluation of IFN-γ-S-specific T-cell response by flow cytometry. Cells from whole blood from HCWs (n = 7) and RA patients (n = 32) were stimulated for 24 h with spike peptide pool, and the percentage of IFN-γ-specific T cells was assessed by flow cytometry. Graphs show the frequency of the **(A)** CD4^+^ and **(B)** CD8^+^ IFN-γ-specific T-cell response (after subtraction of the unstimulated sample) in HCWs and RA patients. Each dot is a different HCW or RA individual, and red lines indicate the median. Mann–Whitney U-test was performed for the statistical analysis, and the p-value ≤0.05 was considered significant. RA, rheumatoid arthritis; HCWs, health care workers.

Flow cytometry analysis revealed that, after 6 months from the first dose of mRNA vaccine, the IFN-γ-specific response is mainly detectable within the CD4^+^ T cells more than within the CD8^+^ T cell compartment, both in HCWs and in RA patients (**Figures 8A, B****)**. The CD4^+^ T-cell response was observed in 43% HCWs (3/7) and in 34% of the RA patients (11/32) (**Figure 8A**). No differences were observed also considering the magnitude of the response in both HCWs and RA patients (median CD4^+^IFN-γ^+^ HCWs: 0.001%, IQR 0.00–0.05 vs. median CD4^+^IFN-γ^+^ RA: 0.00%, IQR: 0.00–0.43, p = 0.5). By contrast, we did not detect a CD8^+^ IFN-γ response in none of the HCWs and only in 12.5% of the RA patients (4/32) (median CD8^+^IFN-γ^+^ HCWs: 0.00%, IQR 0.00–0.00 vs. median CD8^+^IFN-γ^+^ RA: 0.00%, IQR: 0.00–0.00, p = 0.5) (**Figure 8B**). However, both HCWs and RA patients showed an IFN-γ-specific response to the positive control (SEB), providing evidence of the not impaired cytokine production (**Supplementary Figures S5A, B**).

### Decay of the T Cell-Specific Response From T1 to T6

**Figure 9:**
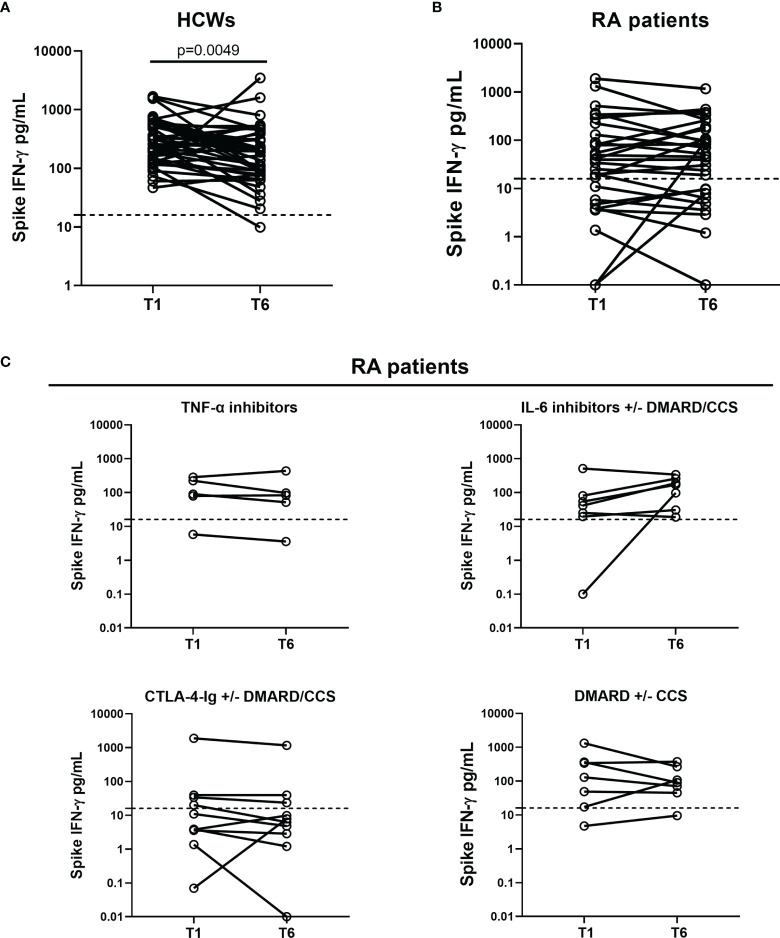
Kinetics of the cell-mediated response induced by SARS-CoV-2 vaccination in HCWs and RA patients. Evaluation of the spike IFN-γ response in 42 HCWs **(A)** and 29 RA patients **(B)** who were longitudinally sampled after 5 weeks (T1) and 6 months (T6) from the first vaccine dose. **(C)** RA patients were stratified in four groups: TNF-α inhibitors (n = 5), IL-6 inhibitors with or without DMARD/CCS (n = 7), CTLA-4-Ig with or without DMARD/CCS (n = 10), and DMARD with or without CCS (n = 7). T-cell response to spike antigen was assessed by measuring IFN-γ levels in plasma harvested from stimulated whole-blood samples. IFN-γ values were reported as median after subtracting the background. Dashed lines indicate the cutoff (spike: 16 pg/ml). Statistical analysis was performed using the Wilcoxon test, and p < 0.05 was considered significant. SARS-CoV-2, severe acute respiratory syndrome coronavirus 2; CCS, corticosteroid; DMARDs, disease-modifying antirheumatic drugs; RA, rheumatoid arthritis; HCWs, health care workers.

Afterward, we evaluated the kinetics of the cellular response comparing the results from the two time points, T1 and T6. In HCWs, a significant reduction of the magnitude of the SARS-CoV-2 IFN-γ-S-specific response was observed at T6 compared to T1 (T1 median: 282.7 pg/ml, IQR: 136.2–570.4 vs. T6 median: 194.3 pg/ml, IQR: 87.0–331.1, p = 0.0049) (**Figure 9A**). By contrast, no significant differences were reported in the entire RA patient cohort, in which the T-cell response remains more stable over time (T1 median: 39.4 pg/ml, IQR: 5.3–177 vs. T6 median: 51.8 pg/ml, IQR: 8.7–180, p = 0.717) (**Figure 9B**). Also stratifying RA patients according to the drug treatment, no significant differences were found: TNF-α inhibitors (T1 median: 89.6 pg/ml, IQR: 42.5–254.6 vs. T6 median: 83 pg/ml, IQR: 28–268, p > 0.999), IL-6 inhibitors (T1 median: 41.3 pg/ml, IQR: 19.5–80.5 vs. T6 median: 170 pg/ml, IQR: 30.6–258, p = 0.297), CTLA-4-Ig (T1 median: 7.42 pg/ml, IQR: 3.03–35.3 vs. T6 median: 7.10 pg/ml, IQR: 2.4–27.6, p = 0.160) and DMARDs±CCSs (T1 median: 130 pg/ml, IQR: 17.2–364 vs. T6 median: 91.0 pg/ml, IQR: 45.3–268, p = 0.578) (**Figure 9C**). A quantitatively increased T-cell response at T6 compared to T1 was found in 11/42 (26.2%) HCWs (median proportion of increase: 69.7%) and in 12/29 (41.4%) RA patients (median proportion of increase: 185%). A reduction of the quantitative T-cell response at T6 compared to T1 was found in 30/42 (71.4%) HCWs (median proportion of reduction: 61.6%) and in 16/29 (55.2%) RA patients (median proportion of reduction: 43.9%). No significant differences were found comparing these two groups (p = 0.2). One subject for each group maintained a stable T-cell response. Finally, among RA patients, 2 subjects initially negative at T1 scored positive at T6.

## Discussion

Currently, vaccination is the most powerful tool to fight the COVID-19 pandemic in the general population and in particular in the fragile populations, such as RA patients. Although evidence is showing suboptimal humoral and cellular immune responses to COVID-19 vaccination in immunocompromised individuals (10, 20, 33, 34), there is a paucity of data regarding the over-time evaluation of both T- and B-cell responses in RA patients.

In this study, we describe the immune response to BNT162b2 vaccine in RA patients vaccinated following ACR indications (19). Accordingly, patients under MTX have interrupted the therapy 1 week after the first and the second dose of the vaccine, while patients under CTLA-4-Ig have interrupted the drug for 1 week before and after only the first dose. The results indicate that neutralizing and anti-RBD IgG titers after BNT162b2 vaccine significantly decrease over time in both HCWs and RA patients. Importantly, patients under TNF-α inhibitors and CTLA-4-Ig showed a significantly lower anti-RBD IgG median titer at T6 compared to HCWs, and this was associated with a reduction in the MBC compartment. Qualitative and quantitative IFN-γ responses were reduced in RA patients at T6 compared to the controls, as reported for the T1 (20). Among the RA patients, those under CTLA-4-Ig treatment were the most affected at T6. However, surprisingly, within the RA patients, we observed subjects who developed a T-cell-specific response at T6 (2/29) (initially scored negative at T1). Moreover, comparing the quantitative T-cell response at T6 vs. T1, we observed an increase of this response in 41.4% of RA patients compared to the 26.2% of HCWs. These results may indicate that the fragile population of RA has a delayed T cell-specific response. Further studies are needed to confirm it. Clinically, BNT162b2 vaccine showed a good safety profile during the 6-month follow-up without serious adverse events and disease relapses. Notably, none of the RA patients had a breakthrough infection.

Regarding the vaccine-induced humoral immunogenicity after 6 months from the completed vaccination, we found that the anti-RBD antibody response was still detectable in all HCWs and in the majority of RA patients, although the titer was significantly reduced in those under TNF-α inhibitors and CTLA-4-Ig compared to HCWs. Neutralizing antibodies scored positive in most HCWs (88%), whereas only 42.8% of RA patients showed a detectable neutralizing activity. Moreover, we found that the anti-RBD and neutralizing antibodies significantly correlated in RA patients and in HCWs. In RA subjects, a moderately significant correlation between the anti-RBD antibody titer and SARS-CoV-2 IFN-γ-S-specific T-cell response was found, highlighting the persistence of a good coordination between the two components of the immune system (35). In contrast, the strong correlation between humoral and T-cell response observed early after vaccination in HCWs (7) was lost over time probably due to the rapid induction of the cell response at T1 in the healthy population that leads to a fast decay over time. Conversely, RA patients mount a more delayed response that leads to a correlation with the antibody response still present at later time points.

Following vaccination, the immune system is activated and produces short-lived plasma cells together with transient structures called germinal centers (GCs), where antigen-activated B cells differentiate into MBCs and into high-affinity long-lived antibody-producing plasma cells. During recalls, MBCs differentiate into long-lived plasma cells or can enter again in the GC reaction, expanding more and differentiate into plasma cells, as the immunological memory protects individuals from reinfections (36–42). Our results show a significant reduction of total MBCs and switched MBC subpopulations in RA patients treated with the CTLA-4-Ig compared to controls; moreover, the frequency of plasma blasts was lower in both CTLA-4-Ig and TNF-α inhibitor-treated patients even if the difference was not significant.

The reduced antibody response to COVID-19 vaccine is likely due to the well-known effect of the CTLA-4-Ig and TNF-α inhibitors on the reduction of the frequency of MBCs in the human peripheral blood (43–45). TNF-α is essential for lymphoid microarchitecture, and an impairment in B-cell function has been reported in RA patients treated with anti-TNF-α agents (45, 46). On the other hand, abatacept (CTLA-4-Ig) by binding with high-affinity CD80/CD86 molecules can affect T costimulation signals expressed by the antigen-presenting cells (47). In this context, a reduction in the humoral immunogenicity to pneumococcal and influenza vaccine has been shown in CTLA-4-Ig-treated patients (48).

Correlates of protection against SARS-CoV-2 infection are not yet established, and, up to now, we evaluated B- and T-cell parameters altogether (49–51). Therefore, looking at the overall specific B- and T-cell responses, patients may be stratified as full responders (both humoral and cellular), not responders, or partial responders (only humoral or T-cell response). Based on this stratification, in our cohort of RA patients, we found that after 6 months from the first dose of SARS-CoV-2 vaccine, 14/35 (40%) RA patients were full responders, 18/35 (51.4%) were partial responders, and only 3 (8.6%) were not responders. Regarding the antibody response, the full responders present both a good anti-RBD antibody titer and a good neutralizing antibody activity, which is one of the best predictors of *in vivo* vaccine efficacy, although it has not been possible so far to obtain a commercial test suggestive of a good correlate of protection (52). Nevertheless, this stratification may help to prioritize the vaccine schedule for RA patients; indeed, the full responders may follow the vaccination schedule as for the healthy individuals. Differently, the partial and/or not responders are very likely to be at higher risk for COVID-19 despite the complete vaccination, underlining the need for a tailored strategy. Although data on the immunogenicity effect of a third booster dose in the immune‐mediated inflammatory disease patients are still lacking, an earlier administration could be hypothesized in not responders or partial responders, as already suggested by others (53).

Recently, we reported that the therapeutic strategy suggested by ACR (19) to interrupt MTX or CTLA-4-Ig (1 week before and after the first dose of the vaccine administration) reduced the negative impact on antibody production previously described (33, 34). Moreover, this strategy was beneficial also for those under CTLA-4-Ig, albeit with a significant lower antibody titer compared to HCWs. Importantly, this temporary therapy interruption did not affect the RA disease activity, as indicated by the stability of the DAS28crp parameter throughout the vaccination period.

In the effort of increasing the chances of complete protection, additional strategies may be explored such as a longer suspension of immunosuppressive agents during the vaccine administration, as well as the switch to drugs that have mechanisms of action with a reduced impact on vaccine immunogenicity (13). The use of a heterologous vaccination may help in enhancing the immune response, as recently reported by a systematic review in the general population, although the immunogenicity was higher in the population vaccinated with ChAdOx1-S followed by BNT162b2 rather than *vice versa* (54). In patients with a “not responder” or “partial responder“ profile, a further clinical option is the use of monoclonal antibodies against SARS-CoV-2-S glycoprotein as primary or secondary prophylaxis prior to or after any known SARS-CoV-2 exposure. Unpublished data from the “prevent” study are likely to support this indication, confirming robust efficacy and long-term prevention (55). Importantly, in parallel, on December 8, 2021, the Food and Drug Administration authorized the use of monoclonal antibodies for pre-exposure prevention of COVID-19 in high-risk population (56).

The main limitation of this study is the small size of the cohort, which may restrict its power, especially for the comparison of the effects of vaccination among the different RA treatments. However, it is important to underline that the enrolled patients are representative of the RA subjects under different immunosuppressive therapies, and that the patients were well characterized, both clinically and immunologically. Finally, the RA cohort and HCWs significantly differed in age, but we previously showed that the immune impairment is associated with the ongoing immunosuppressive therapies more than with the age (20). The main strength of the study is that, for the first time to our knowledge, we deeply characterized over time both humoral and cellular immune responses to BNT162b2 vaccine in RA patients, providing evidence of the kinetics, waning, and drug-induced impairment.

In conclusion, this study shows a significant reduction of the humoral response after 6 months of completed COVID-19 vaccination in both HCWs and RA patients regardless of the immunosuppressive therapy. Interestingly, the T-cell response was significantly decreased in HCWs, whereas it was mostly stable in RA patients. Considering the importance of a coordinated action of both humoral and cell-mediated responses to gain viral protection, our data support the execution of a booster dose of vaccine and a careful timeline of withdrawing some immunosuppressive agents to protect RA individuals from SARS-CoV-2 infection and hospitalization.

## Data Availability Statement

The raw data generated and/or analyzed within the present study are available in our institutional repository (rawdata.inmi.it), subject to registration. The data can be found by selecting the article of interest from a list of articles ordered by year of publication. No charge for granting access to data is required. In the event of a malfunction of the application, the request can be sent directly by e-mail to the Library (biblioteca@inmi.it).

## Ethics Statement

The studies involving human participants were reviewed and approved by the National Institute for Infectious Diseases (INMI) Lazzaro Spallanzani-IRCCS (Approval number 297/2021) and Sant’Andrea University Hospital in Rome (Approval number 318/2021). The patients/participants provided their written informed consent to participate in this study.

## Author Contributions

DG and EN wrote the project to be submitted to the ethical committee. DG, EN, APD, BL, CA, and CC conceived and designed the study. Experiments were performed by CF, AA, SM, FC, RC, GG, VV, AS, FR, and AMGA. CF and AA performed the statistical analysis. APD, BL, GC, RR, SS, GN, GS, GM, and AC enrolled patients and collected clinical data. CF, APD, AA, EN, DG, BL, VP, and FV drafted the article or revised it critically. All authors critically analyzed, discussed, and interpreted data and contributed to the article and approved the submitted version.

## Funding

This work was supported by INMI “Lazzaro Spallanzani” Ricerca Finalizzata COVID-2020-12371675 and Ricerca Corrente on emerging infections both funded by the Italian Ministry of Health and by generous liberal donations funding for COVID-19 research from Esselunga S.p.A., Camera di Commercio, Industria e Artigianato di Roma, Società Numero Blu Servizi S.p.A., Fineco Bank S.p.A., Associazione magistrati della Corte dei conti, and Società Mocerino Frutta Secca s.r.l. (resolutions n°395 of May 25, 2021, n°254 of April 24, 2021, and n°257 of April 14, 2021). The funders were not involved in the study design, collection, analysis, and interpretation of data, the writing of this article, or the decision to submit it for publication.

## Conflict of Interest

APD received fees for educational training or consultancy by Abbvie, Amgen, Novartis, Galapagos, BMS. EN is a member of the advisory board of Gilead, Lilly, and Roche and received fees for educational training by Gilead, Lilly, and Roche. DG is a member of the advisory board of Biomerieux and Eli-Lilly and received fees for educational training or consultancy by Biogen, Cellgene, Diasorin, Janssen, Qiagen, and Quidel.

The remaining authors declare that the research was conducted in the absence of any commercial or financial relationships that could be construed as a potential conflict of interest.

## Publisher’s Note

All claims expressed in this article are solely those of the authors and do not necessarily represent those of their affiliated organizations, or those of the publisher, the editors and the reviewers. Any product that may be evaluated in this article, or claim that may be made by its manufacturer, is not guaranteed or endorsed by the publisher.

## References

[1] Montaldo C Messina F Abbate I Antonioli M Bordoni V Aiello A. Multi-Omics Approach to COVID-19: A Domain-Based Literature Review. J Transl Med (2021) 19:501. doi: 10.1186/s12967-021-03168-8 34876157 PMC8649311

[2] Zhou F Yu T Du R Fan G Liu Y Liu Z. Clinical Course and Risk Factors for Mortality of Adult Inpatients With COVID-19 in Wuhan, China: A Retrospective Cohort Study. Lancet Lond Engl (2020) 395:1054–62. doi: 10.1016/S0140-6736(20)30566-3 PMC7270627 32171076

[3] Nicastri E Petrosillo N Ascoli Bartoli T Lepore L Mondi A Palmieri F. National Institute for the Infectious Diseases “L. Spallanzani”, IRCCS. Recommendations for COVID-19 Clinical Management. Infect Dis Rep (2020) 12:8543. doi: 10.4081/idr.2020.8543 32218915 PMC7097833

[4] Najafi Fard S Petrone L Petruccioli E Alonzi T Matusali G Colavita F. *In Vitro* Models for Studying Entry, Tissue Tropism, and Therapeutic Approaches of Highly Pathogenic Coronaviruses. BioMed Res Int (2021) 2021:8856018. doi: 10.1155/2021/8856018 34239932 PMC8221881

[5] Goletti D Cantini F. Baricitinib Therapy in Covid-19 Pneumonia - An Unmet Need Fulfilled. N Engl J Med (2021) 384:867–9. doi: 10.1056/NEJMe2034982 PMC7944951 33657299

[6] Cantini F Goletti D Petrone L Najafi Fard S Niccoli L Foti R. Immune Therapy, or Antiviral Therapy, or Both for COVID-19: A Systematic Review. Drugs (2020) 80:1929–46. doi: 10.1007/s40265-020-01421-w PMC7568461 33068263

[7] Agrati C Castilletti C Goletti D Meschi S Sacchi A Matusali G. Coordinate Induction of Humoral and Spike Specific T-Cell Response in a Cohort of Italian Health Care Workers Receiving BNT162b2 mRNA Vaccine. Microorganisms (2021) 9:1315. doi: 10.3390/microorganisms9061315 34208751 PMC8235087

[8] Angyal A Longet S Moore SC Payne RP Harding A Tipton T. T-Cell and Antibody Responses to First BNT162b2 Vaccine Dose in Previously Infected and SARS-CoV-2-Naive UK Health-Care Workers: A Multicentre Prospective Cohort Study. Lancet Microbe (2021) 8:e21–31. doi: 10.1016/S2666-5247(21)00275-5 PMC8577846 34778853

[9] Baden LR El Sahly HM Essink B Kotloff K Frey S Novak R. Efficacy and Safety of the mRNA-1273 SARS-CoV-2 Vaccine. N Engl J Med (2021) 384:403–16. doi: 10.1056/NEJMoa2035389 PMC7787219 33378609

[10] Jena A Mishra S Deepak P Kumar-M P Sharma A Patel YI. Response to SARS-CoV-2 Vaccination in Immune Mediated Inflammatory Diseases: Systematic Review and Meta-Analysis. Autoimmun Rev (2022) 21:102927. doi: 10.1016/j.autrev.2021.102927 34474172 PMC8404391

[11] Geisen UM Berner DK Tran F Sümbül M Vullriede L Ciripoi M. Immunogenicity and Safety of Anti-SARS-CoV-2 mRNA Vaccines in Patients With Chronic Inflammatory Conditions and Immunosuppressive Therapy in a Monocentric Cohort. Ann Rheum Dis (2021) 80:1306–11. doi: 10.1136/annrheumdis-2021-220272 PMC8117443 33762264

[12] Simon D Tascilar K Fagni F Krönke G Kleyer A Meder C. SARS-CoV-2 Vaccination Responses in Untreated, Conventionally Treated and Anticytokine-Treated Patients With Immune-Mediated Inflammatory Diseases. Ann Rheum Dis (2021) 80:1312–6. doi: 10.1136/annrheumdis-2021-220461 PMC8103562 33958324

[13] Picchianti Diamanti A Rosado MM Nicastri E Sesti G Pioli C Laganà B. Severe Acute Respiratory Syndrome Coronavirus-2 Infection and Autoimmunity 1 Year Later: The Era of Vaccines. Front Immunol (2021) 12:708848. doi: 10.3389/fimmu.2021.708848 34659200 PMC8515900

[14] Levin EG Lustig Y Cohen C Fluss R Indenbaum V Amit S. Waning Immune Humoral Response to BNT162b2 Covid-19 Vaccine Over 6 Months. N Engl J Med (2021) 385:e84. doi: 10.1056/NEJMoa2114583 34614326 PMC8522797

[15] Bar-On YM Goldberg Y Mandel M Bodenheimer O Freedman L Kalkstein N. Protection of BNT162b2 Vaccine Booster Against Covid-19 in Israel. N Engl J Med (2021) 385:1393–400. doi: 10.1056/NEJMoa2114255 PMC8461568 34525275

[16] Shrotri M Navaratnam AMD Nguyen V Byrne T Geismar C Fragaszy E. Spike-Antibody Waning After Second Dose of BNT162b2 or Chadox1. Lancet Lond Engl (2021) 398:385–7. doi: 10.1016/S0140-6736(21)01642-1 PMC8285117 34274038

[17] Guerrera G Picozza M D’Orso S Placido R Pirronello M Verdiani A. BNT162b2 Vaccination Induces Durable SARS-CoV-2–Specific T Cells With a Stem Cell Memory Phenotype. Sci Immunol (2021) 6:eabl5344. doi: 10.1126/sciimmunol.abl5344 34726470

[18] Aletaha D Neogi T Silman AJ Funovits J Felson DT Bingham CO. 2010 Rheumatoid Arthritis Classification Criteria: An American College of Rheumatology/European League Against Rheumatism Collaborative Initiative. Arthritis Rheum (2010) 62:2569–81. doi: 10.1002/art.27584 20872595

[19] Curtis JR Johnson SR Anthony DD Arasaratnam RJ Baden LR Bass AR. American College of Rheumatology Guidance for COVID-19 Vaccination in Patients With Rheumatic and Musculoskeletal Diseases: Version 3. Arthritis Rheumatol Hoboken NJ (2021) 73:e60–75. doi: 10.1002/art.41928 PMC8426685 34346564

[20] Picchianti-Diamanti A Aiello A Laganà B Agrati C Castilletti C Meschi S. ImmunosuppressiveTherapies Differently Modulate Humoral- and T-Cell-Specific Responses to COVID-19 mRNA Vaccine in Rheumatoid Arthritis Patients. Front Immunol (2021) 12:740249. doi: 10.3389/fimmu.2021.740249 34594343 PMC8477040

[21] Matusali G Colavita F Lapa D Meschi S Bordi L Piselli P. SARS-CoV-2 Serum Neutralization Assay: A Traditional Tool for a Brand-New Virus. Viruses (2021) 13:655. doi: 10.3390/v13040655 33920222 PMC8069482

[22] Carsetti R Zaffina S Piano Mortari E Terreri S Corrente F Capponi C. Different Innate and Adaptive Immune Responses to SARS-CoV-2 Infection of Asymptomatic, Mild, and Severe Cases. Front Immunol (2020) 11:610300. doi: 10.3389/fimmu.2020.610300 33391280 PMC7772470

[23] Aiello A Najafi Fard S Petruccioli E Petrone L Vanini V Farroni C. Spike Is the Most Recognized Antigen in the Whole-Blood Platform in Both Acute and Convalescent COVID-19 Patients. Int J Infect Dis IJID Off Publ Int Soc Infect Dis (2021) 106:338–47. doi: 10.1016/j.ijid.2021.04.034 PMC8045417 33864921

[24] Tortorella C Aiello A Gasperini C Agrati C Castilletti C Ruggieri S. Humoral- and T-Cell-Specific Immune Responses to SARS-CoV-2 mRNA Vaccination in Patients With MS Using Different Disease-Modifying Therapies. Neurology (2021) 98:e541–54. doi: 10.1212/WNL.0000000000013108 PMC8826460 34810244

[25] Murugesan K Jagannathan P Pham TD Pandey S Bonilla HF Jacobson K. Interferon-γ Release Assay for Accurate Detection of Severe Acute Respiratory Syndrome Coronavirus 2 T-Cell Response. Clin Infect Dis Off Publ Infect Dis Soc Am (2021) 73:e3130–2. doi: 10.1093/cid/ciaa1537 PMC7665338 33035306

[26] Riou C Schäfer G du Bruyn E Goliath RT Stek C Mou H. Rapid, Simplified Whole Blood-Based Multiparameter Assay to Quantify and Phenotype SARS-CoV-2 Specific T Cells. Eur Respir J (2021) 59:2100285. doi: 10.1183/13993003.00285-2021 PMC8215505 34140294

[27] Petruccioli E Najafi Fard S Navarra A Petrone L Vanini V Cuzzi G. Exploratory Analysis to Identify the Best Antigen and the Best Immune Biomarkers to Study SARS-CoV-2 Infection. J Transl Med (2021) 19:272. doi: 10.1186/s12967-021-02938-8 34174875 PMC8235902

[28] Petrone L Petruccioli E Vanini V Cuzzi G Najafi Fard S Alonzi T. A Whole Blood Test to Measure SARS-CoV-2-Specific Response in COVID-19 Patients. Clin Microbiol Infect Off Publ Eur Soc Clin Microbiol Infect Dis (2021) 27:286.e7–286.e13. doi: 10.1016/j.cmi.2020.09.051 PMC7547312 33045370

[29] Petrone L Petruccioli E Alonzi T Vanini V Cuzzi G Najafi Fard S. *In-Vitro* Evaluation of the Immunomodulatory Effects of Baricitinib: Implication for COVID-19 Therapy. J Infect (2021) 82:58–66. doi: 10.1016/j.jinf.2021.02.023 33639176 PMC7904476

[30] Kagina BM Mansoor N Kpamegan EP Penn-Nicholson A Nemes E Smit E. Qualification of a Whole Blood Intracellular Cytokine Staining Assay to Measure Mycobacteria-Specific CD4 and CD8 T Cell Immunity by Flow Cytometry. J Immunol Methods (2015) 417:22–33. doi: 10.1016/j.jim.2014.12.003 25523923 PMC4339399

[31] Nemes E Hesseling AC Tameris M Mauff K Downing K Mulenga H. Safety and Immunogenicity of Newborn MVA85A Vaccination and Selective, Delayed Bacille Calmette-Guerin for Infants of Human Immunodeficiency Virus-Infected Mothers: A Phase 2 Randomized, Controlled Trial. Clin Infect Dis Off Publ Infect Dis Soc Am (2018) 66:554–63. doi: 10.1093/cid/cix834 PMC5849090 29028973

[32] Roederer M. How Many Events Is Enough? Are You Positive? Cytom Part J Int Soc Anal Cytol (2008) 73:384–5. doi: 10.1002/cyto.a.20549 18307257

[33] Furer V Eviatar T Zisman D Peleg H Paran D Levartovsky D. Immunogenicity and Safety of the BNT162b2 mRNA COVID-19 Vaccine in Adult Patients With Autoimmune Inflammatory Rheumatic Diseases and in the General Population: A Multicentre Study. Ann Rheum Dis (2021) 80:1330–8. doi: 10.1136/annrheumdis-2021-220647 34127481

[34] Haberman RH Herati R Simon D Samanovic M Blank RB Tuen M. Methotrexate Hampers Immunogenicity to BNT162b2 mRNA COVID-19 Vaccine in Immune-Mediated Inflammatory Disease. Ann Rheum Dis (2021) 80:1339–44. doi: 10.1136/annrheumdis-2021-220597 PMC8219484 34035003

[35] Sette A Crotty S. Adaptive Immunity to SARS-CoV-2 and COVID-19. Cell (2021) 184:861–80. doi: 10.1016/j.cell.2021.01.007 PMC7803150 33497610

[36] Akkaya M Kwak K Pierce SK. B Cell Memory: Building Two Walls of Protection Against Pathogens. Nat Rev Immunol (2020) 20:229–38. doi: 10.1038/s41577-019-0244-2 PMC7223087 31836872

[37] Grimsholm O Piano Mortari E Davydov AN Shugay M Obraztsova AS Bocci C. The Interplay Between CD27dull and CD27bright B Cells Ensures the Flexibility, Stability, and Resilience of Human B Cell Memory. Cell Rep (2020) 30:2963–2977.e6. doi: 10.1016/j.celrep.2020.02.022 32130900

[38] Dunn-Walters DK Stewart AT Sinclair EL Serangeli I. Age-Related Changes in B Cells Relevant to Vaccine Responses. Interdiscip Top Gerontol Geriatr (2020) 43:56–72. doi: 10.1159/000504479 32305972

[39] Farroni C Marasco E Marcellini V Giorda E Valentini D Petrini S. Dysregulated miR-155 and miR-125b Are Related to Impaired B-Cell Responses in Down Syndrome. Front Immunol (2018) 9:2683. doi: 10.3389/fimmu.2018.02683 30515165 PMC6255899

[40] Valentini D Marcellini V Bianchi S Villani A Facchini M Donatelli I. Generation of Switched Memory B Cells in Response to Vaccination in Down Syndrome Children and Their Siblings. Vaccine (2015) 33:6689–96. doi: 10.1016/j.vaccine.2015.10.083 26518399

[41] Tsang JS Schwartzberg PL Kotliarov Y Biancotto A Xie Z Germain RN. Global Analyses of Human Immune Variation Reveal Baseline Predictors of Postvaccination Responses. Cell (2014) 157:499–513. doi: 10.1016/j.cell.2014.03.031 24725414 PMC4139290

[42] Pulendran B Ahmed R. Immunological Mechanisms of Vaccination. Nat Immunol (2011) 12:509–17. doi: 10.1038/ni.2039 PMC3253344 21739679

[43] Scarsi M Paolini L Ricotta D Pedrini A Piantoni S Caimi L. Abatacept Reduces Levels of Switched Memory B Cells, Autoantibodies, and Immunoglobulins in Patients With Rheumatoid Arthritis. J Rheumatol (2014) 41:666–72. doi: 10.3899/jrheum.130905 24584924

[44] Sanz I Wei C Lee FE-H Anolik J. Phenotypic and Functional Heterogeneity of Human Memory B Cells. Semin Immunol (2008) 20:67–82. doi: 10.1016/j.smim.2007.12.006 18258454 PMC2440717

[45] Anolik JH Ravikumar R Barnard J Owen T Almudevar A Milner ECB. Cutting Edge: Anti-Tumor Necrosis Factor Therapy in Rheumatoid Arthritis Inhibits Memory B Lymphocytes *via* Effects on Lymphoid Germinal Centers and Follicular Dendritic Cell Networks. J Immunol (2008) 180:688–92. doi: 10.4049/jimmunol.180.2.688 18178805

[46] Picchianti Diamanti A Rosado MM Scarsella M Germano V Giorda E Cascioli S. Abatacept (Cytotoxic T Lymphocyte Antigen 4-Immunoglobulin) Improves B Cell Function and Regulatory T Cell Inhibitory Capacity in Rheumatoid Arthritis Patients Non-Responding to Anti-Tumour Necrosis Factor-α Agents. Clin Exp Immunol (2014) 177:630–40. doi: 10.1111/cei.12367 PMC4137847 24773026

[47] Moreland L Bate G Kirkpatrick P. Abatacept. Nat Rev Drug Discov (2006) 5:185–6. doi: 10.1038/nrd1989 16557658

[48] Rondaan C Furer V Heijstek MW Agmon-Levin N Bijl M Breedveld FC. Efficacy, Immunogenicity and Safety of Vaccination in Adult Patients With Autoimmune Inflammatory Rheumatic Diseases: A Systematic Literature Review for the 2019 Update of EULAR Recommendations. RMD Open (2019) 5:e001035. doi: 10.1136/rmdopen-2019-001035 31565247 PMC6744079

[49] Dan JM Mateus J Kato Y Hastie KM Yu ED Faliti CE. Immunological Memory to SARS-CoV-2 Assessed for Up to 8 Months After Infection. Science (2021) 371:eabf4063. doi: 10.1126/science.abf4063 33408181 PMC7919858

[50] Goletti D Petrone L Manissero D Bertoletti A Rao S Ndunda N. The Potential Clinical Utility of Measuring Severe Acute Respiratory Syndrome Coronavirus 2-Specific T-Cell Responses. Clin Microbiol Infect Off Publ Eur Soc Clin Microbiol Infect Dis (2021) 27:1784–9. doi: 10.1016/j.cmi.2021.07.005 PMC8272618 34256141

[51] Ferraccioli G Gremese E Goletti D Petrone L Cantini F Ugel S. Immune-Guided Therapy of COVID-19. Cancer Immunol Res (2022):canimm.0675.2021. doi: 10.1158/2326-6066.CIR-21-0675 35074758

[52] Khoury DS Cromer D Reynaldi A Schlub TE Wheatley AK Juno JA. Neutralizing Antibody Levels Are Highly Predictive of Immune Protection From Symptomatic SARS-CoV-2 Infection. Nat Med (2021) 27:1205–11. doi: 10.1038/s41591-021-01377-8 34002089

[53] Ferri C Ursini F Gragnani L Raimondo V Giuggioli D Foti R. Impaired Immunogenicity to COVID-19 Vaccines in Autoimmune Systemic Diseases. High Prevalence of Non-Response in Different Patients’ Subgroups. J Autoimmun (2021) 125:102744. doi: 10.1016/j.jaut.2021.102744 34781162 PMC8577991

[54] Ho T-C Chen Y-MA Chan H-P Chang C-C Chuang K-P Lee C-H. The Effects of Heterologous Immunization With Prime-Boost COVID-19 Vaccination Against SARS-CoV-2. Vaccines (2021) 9:1163. doi: 10.3390/vaccines9101163 34696271 PMC8537265

[55] New Analyses of Two AZD7442 COVID-19 Phase III Trials in High-Risk Populations Confirm Robust Efficacy and Long-Term Prevention. Available at: https://www.astrazeneca.com/media-centre/press-releases/2021/new-analyses-of-two-azd7442-covid-19-phase-iii-trials-in-high-risk-populations-confirm-robust-efficacy-and-long-term-prevention.html (Accessed December 30, 2021).

[56] Commissioner O of the. Coronavirus (COVID-19) Update: FDA Authorizes New Long-Acting Monoclonal Antibodies for Pre-Exposure Prevention of COVID-19 in Certain Individuals. FDA (2021). Available at: https://www.fda.gov/news-events/press-announcements/coronavirus-covid-19-update-fda-authorizes-new-long-acting-monoclonal-antibodies-pre-exposure (Accessed December 30, 2021).

