# Supplementary material for: Kinetics of the B- and T-Cell Immune Responses After 6 Months From SARS-CoV-2 mRNA Vaccination in Patients With Rheumatoid Arthritis
Source: Front Immunol. 2022 Feb 28;13:846753. doi: 10.3389/fimmu.2022.846753 (PMC8924958; doi:10.3389/fimmu.2022.846753)
Supplement: Supplementary file 1 [file DataSheet_1.docx]

Supplementary Material

1. **Supplementary Table**

**Supplementary Table S1.** List of antibodies and reagents for flow cytometry analysis

|  | **Antibody/Reagent** | **Cat#** | **From** |
| --- | --- | --- | --- |
| **T-cell Panel** | Fixable Viability stain 700 APC-R700 | 564997 | BD Bioscience |
|  | CD3 V450 | 560365 | BD Bioscience |
|  | CD4 BV711 | 563028 | BD Bioscience |
|  | CD8 APC-H7 | 641400 | BD Bioscience |
|  | IFNg BV510 | 563287 | BD Bioscience |
| **B-cell Panel** | CD45 V500 | B-cell Tube    626220 | BD Bioscience |
|  | CD19 PE-Cy7 |  |  |
|  | CD27 APC |  |  |
|  | CD38 PerCP-Cy5.5 |  |  |
|  | IgM FITC |  |  |
|  | IgG APC-H7 |  |  |
|  | IgD V450 |  |  |
| ***in vitro* stimulation and flow cytometry reagents** | purified human antiCD28 | 555726 | BD Bioscience |
|  | purified human antiCD49d | 555502 | BD Bioscience |
|  | Brefeldin A | S-B7651-5MG | SIGMA-ALDRICH |
|  | BD FACSLysing Solution | 349202 | BD Bioscience |
|  | BD Horizon Brilliant Stain buffer | 563794 | BD Bioscience |
|  | Perm Wash (Cytofix/Cytoperm solution kit) | 554714 | BD Bioscience |
|  | Formaldehyde | 415666 | Carlo Erba |

1. **Supplementary Figures**

**Supplementary Figure S1**

**
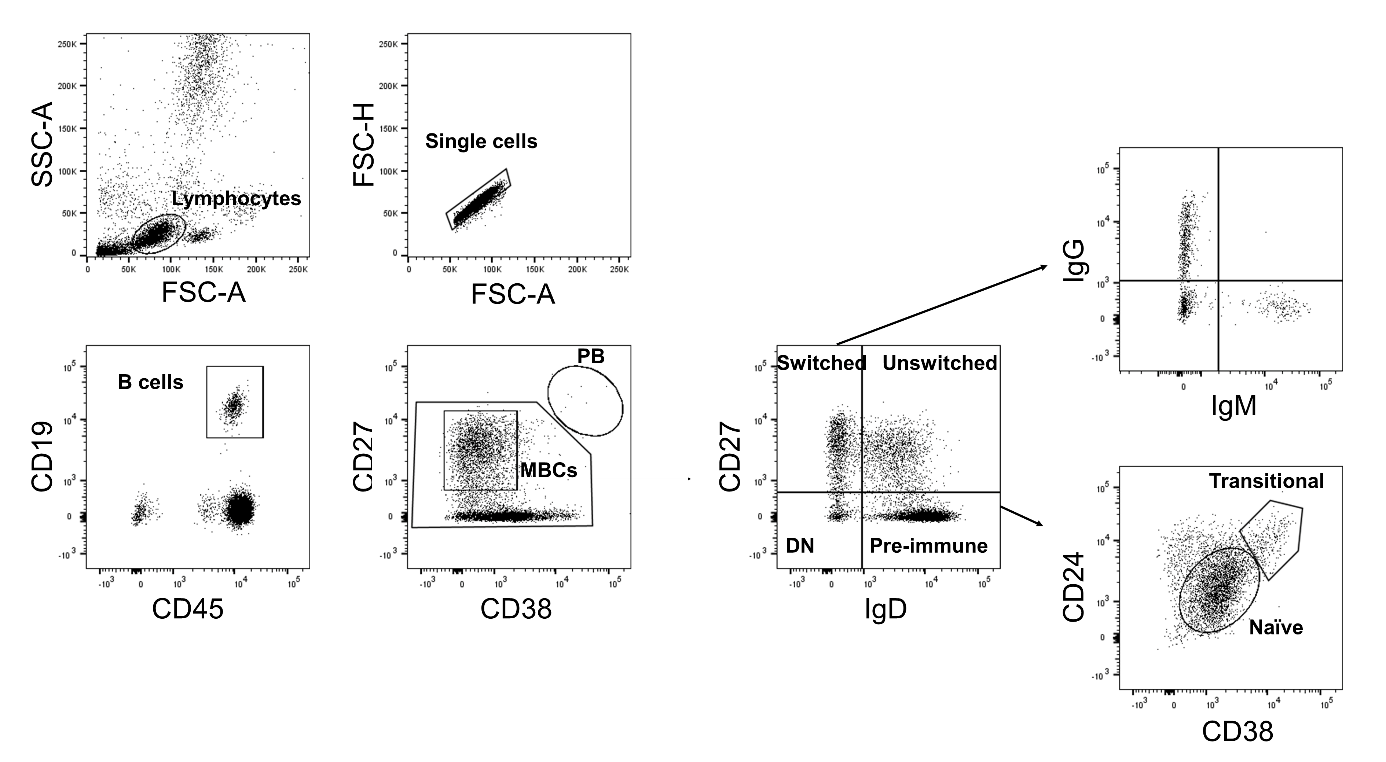
**

**Supplementary Figure S1. B-cell flow cytometry gating strategy**. (**A**) B-cell subpopulations were gated according to the expression of surface markers as described in a representative HCW subject. For the staining of B-cells, the B-cell tube (BD Bioscience) was used and includes: CD19, CD45, CD24, CD27, CD38, IgD, IgM, IgG. B cells were gated as CD19^+^ CD45^+^. Footnotes: MBCs: memory B cells; PB: plasma blast; DN: double negative.

**Supplementary Figure S2**

**
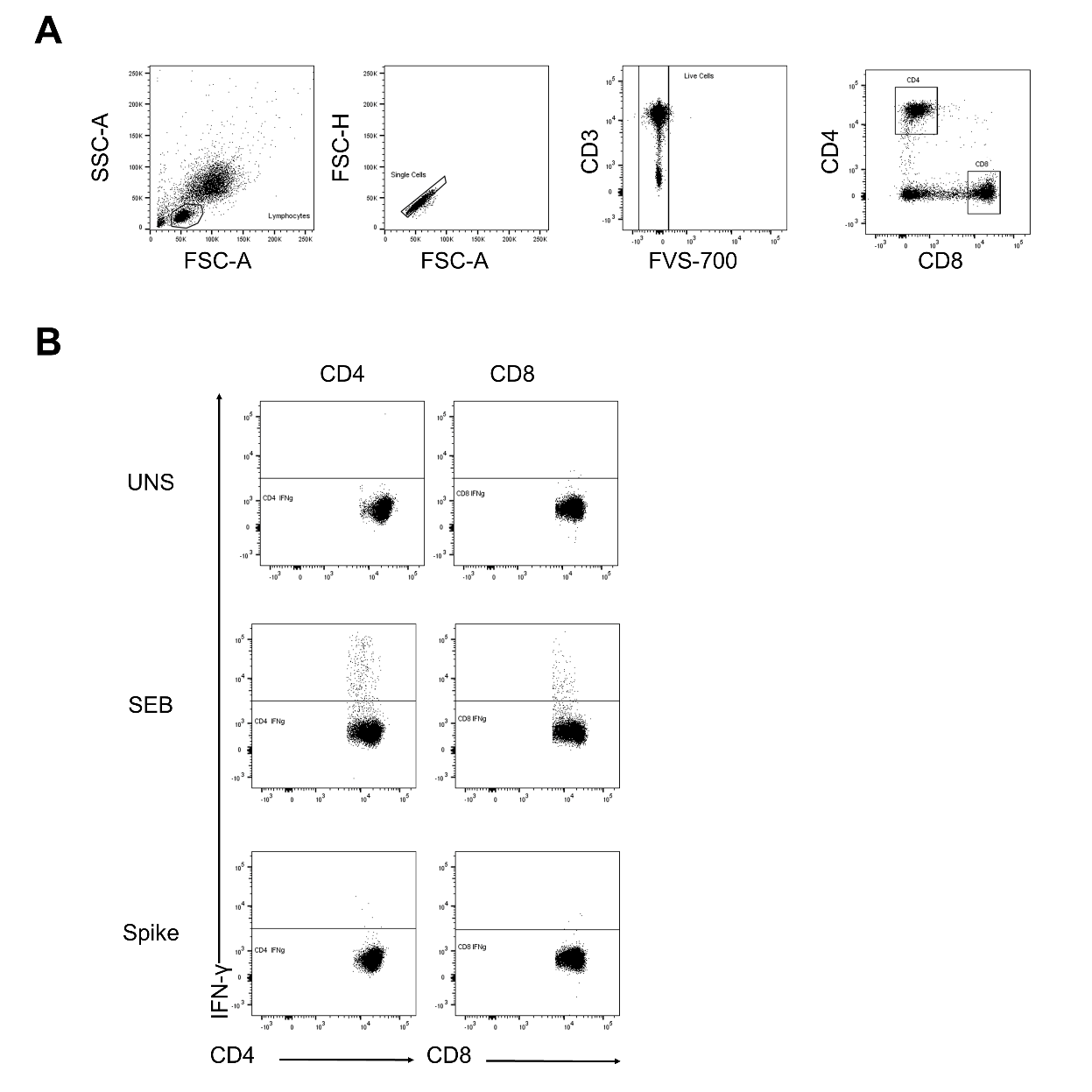
**

**Supplementary Figure S2. T-cell flow cytometry gating strategy**. (**A**) T-cell subpopulations were gated according to the expression of surface markers as described in a representative HCW subject. (**B**) CD4^+^ T-cell-specific response (left plots) and CD8^+^ T-cell-specific response (right plots) were gated as indicated. The comparison with the unstimulated condition is shown (top plots). Footnotes: IFN, Interferon; RA, rheumatoid arthritis; HCWs, health care workers; SEB, Staphylococcal Enterotoxin B.

**Supplementary Figure S3**


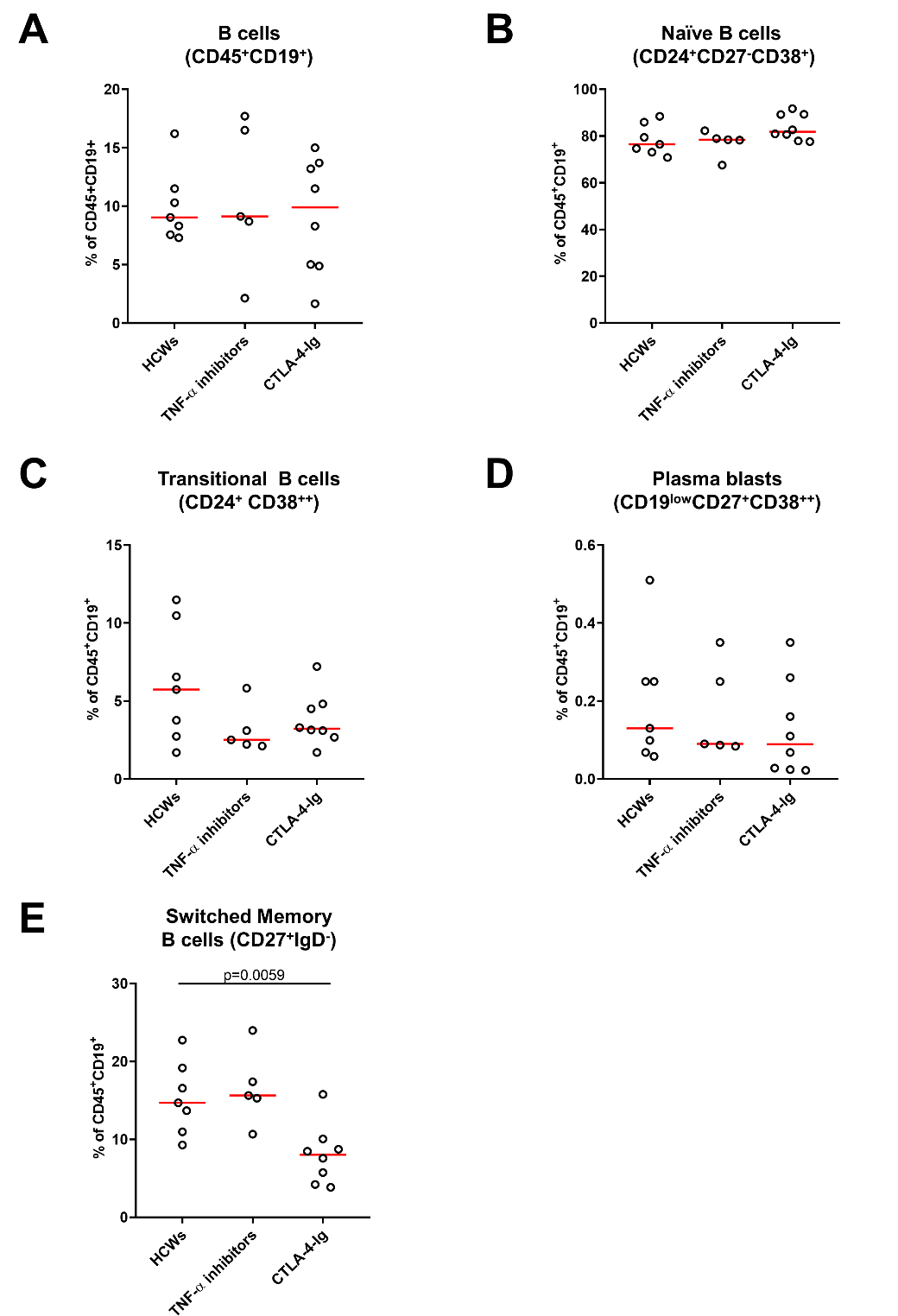


**Supplementary Figure S3. Evaluation of B-cell phenotype of rheumatoid arthritis patients** **by flow cytometry.** B-cell phenotype of HCWs (n=7) and RA patients (n=13) was evaluated by flow cytometry. Within RA patients, 5 subjects were treated with TNF-α inhibitors and 8 with CTLA-4 inhibitors. Frequency of B cells (**A**), naïve B cells (CD24^+^CD27^-^CD38^+^) (**B**), transitional B cells (CD24^+^CD38^++^) (**C**), plasma blasts (CD19^low^CD27^+^CD38^++^) (**D**) and switched memory B cells (CD27^+^IgD^-^) (**E**) were reported. Graphs indicate the percentage of total CD19^+^CD45^+^ B cells. No significant differences were observed compared to HCWs, except for patients treated with CTLA-4 inhibitors (p=0.0033). Each dot represents an individual and black lines represent medians. Statistical analysis was performed using the Mann-Whitney U-test with Bonferroni correction (p≤0.025). Footnotes: RA, rheumatoid arthritis; HCWs, health care workers.

**Supplementary Figure S4**


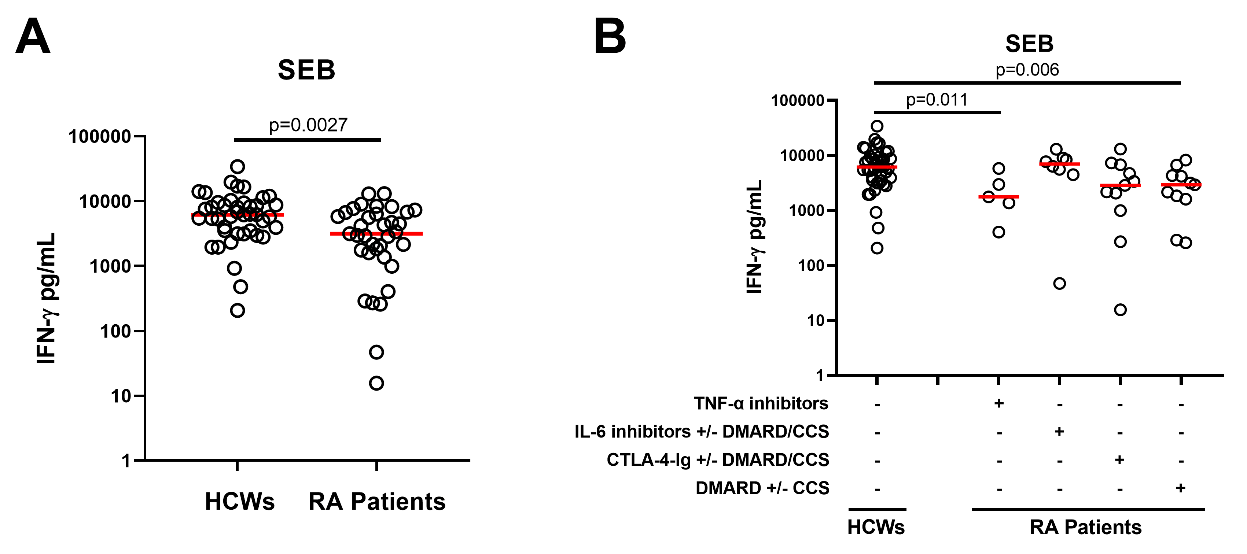


**Supplementary Figure S4. IFN-γ response to Staphylococcal Enterotoxin B (SEB).** Evaluation of the IFN-γ-Spike-specific response in the total of HCWs (n=49) and RA patients (n=35) analysed after 6 months from vaccination (**A**). (**B**) RA patients were also stratified in four groups: TNF-α inhibitors (n=5), IL-6 inhibitors with or w/o DMARD/CCS (n=8), CTLA-4-Ig with or w/o DMARD/CCS (n=11) and DMARD with or w/o CCS (n=11). T-cell response to SEB stimulus was assessed by quantifying IFN-γ levels in the plasma harvested from stimulated whole-blood samples. IFN-γ values were reported after subtracting the background. Statistical analysis was performed using Mann-Whitney U-test (p≤0.05) (**A**) with Bonferroni correction (p≤0.0125) (**B**). Footnotes: CCS, Corticosteroid; DMARDs, Disease Modifying Anti-Rheumatic Drugs; RA, rheumatoid arthritis; HCWs, health care workers.

**Supplementary Figure S5**


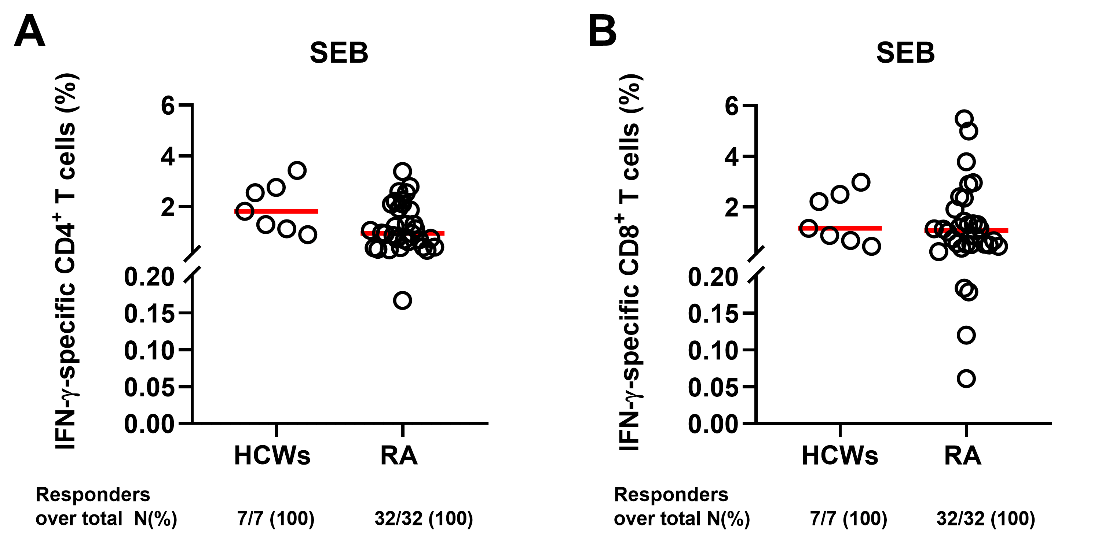


**Supplementary Figure S5. CD4^+^ and CD8^+^ T-cell response to SEB**. Cells from whole blood from HCWs (n=7) and RA patients (n=32) were stimulated in vitro for 24h with SEB, as positive control. IFN-γ response to SEB stimulus was detected in both HCWs and RA individuals in CD4^+^ T cells (A) and CD8^+^ T cells (B). Each dot represents a different HCW or RA individual. Red lines represent the median. Statistical analysis was performed using the Mann-Whitney U-test and p value was considered significant if ≤0.05. ** p=0.003. Footnotes: IFN, Interferon; RA, rheumatoid arthritis; HCWs, health care workers; SEB, Staphylococcal Enterotoxin B.
